# The Evolutionary Trajectory and Prognostic Value of GITR+ Tregs Reprogramed by Tumor‐Intrinsic PD‐1/c‐MET Signaling in Pancreatic Cancer

**DOI:** 10.1002/advs.202500806

**Published:** 2025-07-17

**Authors:** Jiande Han, Hanlin Yin, Taochen He, Junyi He, Zhenlai Jiang, Qiangda Chen, Zhihang Xu, Yuqi Xie, Yaolin Xu, Haibo Wang, Wenquan Wang, Wenchuan Wu, Yun Jin, Wenhui Lou, Jun Yu, Ning Pu, Liang Liu

**Affiliations:** ^1^ Department of Pancreatic Surgery Zhongshan Hospital Fudan University Shanghai 200032 China; ^2^ Cancer Center Zhongshan Hospital Fudan University Shanghai 200032 China; ^3^ Department of Hepatobiliary Pancreatic Surgery The First Affiliated Hospital of Fujian Medical University Fuzhou 350005 China; ^4^ Department of Hepatobiliary and Pancreatic Surgery The First People's Hospital of Yunnan Province The Affiliated Hospital of Kunming University of Science and Technology Kunming 650500 China; ^5^ Pancreas Center Tianjin Medical University Cancer Institute and Hospital National Clinical Research Center for Cancer State Key Laboratory of Druggability Evaluation and Systematic Translational Medicine Tianjin Key Laboratory of Digestive Cancer Tianjin's Clinical Research Center for Cancer Tianjin 300060 China; ^6^ Medical College of Yangzhou University Yangzhou 225002 China; ^7^ Key Laboratory of Gastrointestinal Cancer (Fujian Medical University) Ministry of Education School of Basic Medical Sciences Fujian Medical University 1 Xue Fu North Road Fuzhou 350122 China

**Keywords:** GITR, MET, pancreatic ductal adenocarcinoma, prognosis, regulatory T cells, tumor‐intrinsic PD‐1

## Abstract

Tumor‐intrinsic programmed cell death 1 (PD‐1) has been shown to activate the mesenchymal epithelial transition factor (MET) pathway via its phosphorylation in pancreatic ductal adenocarcinoma (PDAC). However, the immunoregulatory consequences of MET activation remain poorly understood. Herein, a significant positive correlation between phosphorylated MET (p‐MET) and tumor‐intrinsic PD‐1 is verified, both of which are independently associated with adverse prognosis. Elevated p‐MET levels correlated with diminished CD8^+^ T cell cytotoxicity and increased regulatory T cell (Treg) infiltration. Single‐cell RNA sequencing revealed MET activation selectively drives the accumulation of intratumoral GITR⁺ Tregs—a distinct effector Treg subset with potent immunosuppressive function and high prognostic relevance. Compared to KLF2⁺ naïve Tregs, GITR⁺ Tregs exhibited an activated phenotype and enhanced expression of immunoregulatory markers. Subgroup analysis further demonstrated that elevated GITR⁺ Treg infiltration diminished the prognostic utility of serum CA19‐9, underscoring the immunosuppressive dominance of this Treg subset. Mechanistically, MET–IL‐23–STAT4 axis orchestrates GITR⁺ Treg‐mediated immune evasion in PDAC. In vivo, MET inhibition and GITR agonism synergize to enhance antitumor immunity in an orthotopic PDAC model. Collectively, these findings highlight MET signaling and GITR⁺ Tregs as actionable targets to counteract immune evasion and improve the efficacy of immunotherapeutic strategies in PDAC.

## Graphical abstract

In pancreatic ductal adenocarcinoma (PDAC), tumor‐intrinsic PD‐1 signaling activates the MET pathway, leading to the establishment of an immunosuppressive tumor microenvironment (TME). This MET‐driven signaling cascade promotes the selective accumulation of GITR^+^ regulatory T cells (Tregs), a highly immunosuppressive subset. Mechanistically, MET activation facilitates GITR^+^ Treg differentiation via the IL‐23/STAT4 axis. Importantly, co‐targeting MET and GITR yields synergistic antitumor effects in a murine PDAC model, highlighting a promising combinatorial strategy to overcome immunosuppression and improve therapeutic outcomes in PDAC.

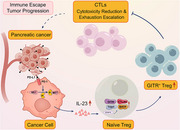

## 1 Introduction

Pancreatic ductal adenocarcinoma (PDAC) is an exceptionally aggressive cancer with a low global incidence but remains one of the most lethal malignancies, exhibiting a five‐year survival rate below 13% and increasing in prevalence in recent years.^[^ ^1^ ^]^ Despite significant advancements in immunotherapy that have enhanced outcomes for various cancers, its efficacy in treating PDAC remains limited.^[^ ^2^, ^3^ ^]^ The reasons for this are multifaceted, with one major factor being the highly immunosuppressive tumor microenvironment (TME) of PDAC.^[^ ^4^ ^]^ This TME is rich in components such as tumor‐associated fibroblasts, regulatory T cells (Tregs), and M2 macrophages, which facilitate immune evasion by cancer cells.^[^ ^4^, ^5^ ^]^ These immunosuppressive elements are primarily driven by interactions between tumor cells and the tumor immune microenvironment (TIME), including pathways like the PD‐1/PD‐L1 axis, CXCL1/CXCR2 axis, and TGF‐β/TGF‐βR axis.^[^ ^6^, ^7^ ^]^ Targeting and modulating these interactions or the immunosuppressive components within the TME presents promising strategies for enhancing the effectiveness of immunotherapy in PDAC.

Programmed Cell Death 1 (PD‐1) is a well‐known immune checkpoint primarily expressed on the surface of effector T cells, where it plays a critical role in regulating immune responses and is a key target in cancer immunotherapy.^[^ ^8^ ^]^ However, PD‐1 is also expressed in tumor cells, a phenomenon that is often overlooked.^[^ ^9^ ^]^ Our previous research has shown that high levels of tumor‐intrinsic PD‐1 promote tumor cell proliferation by activating the Hippo signaling pathway.^[^ ^10^ ^]^ Additionally, a research team in the United States discovered that in pancreatic cancer, activation of tumor‐intrinsic PD‐1 enhances the phosphorylation of the Mesenchymal‐Epithelial Transition factor (MET), thereby facilitating the epithelial‐mesenchymal transition (EMT) in tumor cells.^[^ ^11^ ^]^

MET is a receptor tyrosine kinase predominantly expressed in barrier cells, such as epithelial and endothelial cells. It is essential for various physiological processes, including cell proliferation, survival and morphogenesis.^[^ ^12^ ^]^ In the context of cancer, MET is recognized as a key oncogene driver. Abnormal amplification, mutation, or fusion of the MET gene in cancer cells leads to the aberrant activation of the MET pathway. This pathway can be activated not only by its primary ligand, Hepatocyte Growth Factor (HGF), but also through interactions with other ligands.^[^ ^13^ ^]^ In several types of cancers, activation of MET has been well‐documented to promote tumor progression by creating an immunosuppressive TIME.^[^ ^14^, ^15^, ^16^ ^]^ However, the specific effects of MET activation on the TIME in PDAC remain poorly understood and warrant further investigation. Understanding these mechanisms is essential for developing more effective immunotherapeutic strategies for PDAC.

In this study, we identified a positive correlation between tumor‐intrinsic PD‐1 expression and MET activation in PDAC. We further examined the relationship between the immunosuppressive TIME and MET activation, revealing a significant association with the accumulation of GITR^+^ Tregs. Notably, GITR^+^ Tregs, previously unreported in PDAC, represent a highly immunosuppressive subset of Tregs and serve as important prognostic indicators for PDAC outcomes. Our findings indicate that tumor‐intrinsic PD‐1‐mediated MET activation promotes the accumulation of immunosuppressive GITR^+^ Tregs through IL‐23‐mediated STAT4 phosphorylation in Tregs. This pathway contributes to the establishment of an immunosuppressive TME in PDAC. Preclinical evidence from our models suggest that therapeutic targeting MET or GITR^+^ Treg populations may offer an effective strategy to modulate immune suppression in PDAC and enhance the efficacy of immunotherapy.

## 2 Results

### 2.1 Tumor‐Intrinsic PD‐1 Serves as a Predictor for PDAC Prognosis and Correlates with MET Activation

Our previous work identified tumor‐intrinsic PD‐1 as a promotor of tumor progression and an independent prognostic factor in PDAC.^[^ ^10^ ^]^ To further validate its clinical relevance, we analyzed an independent cohort of 29 PDAC patients. This cohort consisted of 55.2% males with a median age of 64.7 years. According to the AJCC 8th TNM staging system, 34.5% of patients were stage I, 44.8% stage II, and 20.7% stage III (Table S1, Supporting Information). Kaplan‐Meier survival analysis demonstrated that high expression of tumor‐intrinsic PD‐1, determined by IHC staining, was significantly associated with shorter OS (20.0 months vs. N.A., p = 0.017, HR = 3.093, 95% CI: 0.989‐9.672, Figure S1A, Supporting Information) and reduced RFS (9.0 months vs. 29.0 months, p = 0.003, HR = 3.463, 95% CI: 1.278‐9.385, Figure S1B, Supporting Information). These findings were independently confirmed in the TCGA cohort (Figure S1C,D, Supporting Information). To ensure PD‐1 expression was derived from tumor cells, and not from infiltrating immune or stromal components, we selected samples with high tumor purity (ABSOLUTE‐predicted purity > 0.33), as validated in prior studies.^[^ ^17^ ^]^ Within these high‐purity TCGA samples, elevated tumor‐intrinsic PD‐1 expression remained significantly associated with worse outcomes, including shorter OS (15.7 months vs. 37.7 months, p = 0.011, HR = 2.032, 95% CI:1.207‐3.419, Figure S1C, Supporting Information) and RFS (12.4 months vs. 18.5 months, p = 0.020, HR = 1.863, 95% CI: 1.121‐3.094, Figure S1D, Supporting Information). These results are consistent with our previous study,^[^ ^10^ ^]^ and further establish tumor‐intrinsic PD‐1 as a reliable prognostic biomarker in PDAC.

**Figure 1:**
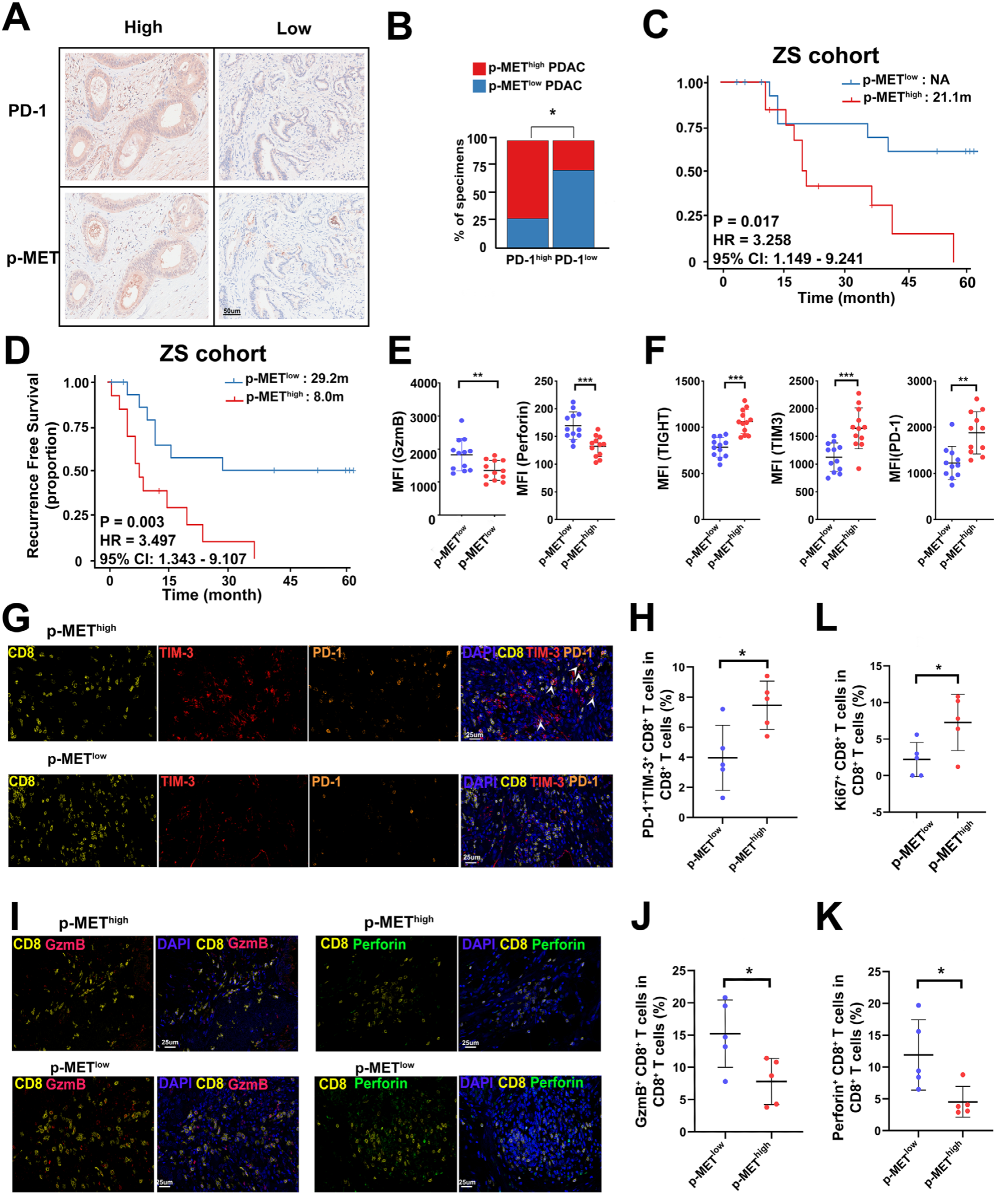
MET activation is associated with tumor intrinsic PD‐1 expression and an immunosuppressive TME in PDAC. A) Representative immunohistochemistry image of PDAC tissues showing high or low tumor‐intrinsic PD‐1 and p‐MET expression. B) Percentages of p‐MET^high^ and p‐MET^low^ specimensin patients with high or low tumor‐intrinsic PD‐1 expression group. ^*^ *p *< 0.05. C,D) Kaplan‐Meier curves for OS (C) and RFS (D) in PDAC patients with high or low p‐MET expression in ZS cohort (*n* = 29). E) Quantification analysis of flow cytometry to compare cytotoxic cytokines in tumor infiltrating CD8^+^ T cells between PDAC patients with high or low p‐MET expression, see also in Figure S3A (Supporting Information). *n* = 12 per group. ^*^ *p* < 0.05; ^***^ *p* < 0.001. F) Quantification analysis of flow cytometry to compare exhausted markers in tumor infiltrating CD8^+^ T cells between PDAC patients with high or low p‐MET expression, see also in Figure S3B (Supporting Information). *n* = 12 per group. ^**^ *p* < 0.01; ^***^ *p* < 0.001. G) Representative immunofluorescence staining images for p‐MET and tumor‐infiltrating TIM‐3^+^PD‐1^+^ CD8^+^ T cells in p‐MET high and p‐MET low tumors. While arrow refers to CD8^+^PD‐1^+^TIM‐3^+^ cells. H) Quantification of TIM‐3^+^PD‐1^+^CD8^+^ T cells infiltration in p‐MET^high^ and p‐MET^low^ tumors. *n* = 5 per group. ^*^ *p* < 0.05. (I) Representative immunofluorescence staining images for p‐MET and tumor‐infiltrating GzmB^+^CD8^+^ T cells or Perforin^+^CD8^+^ T cells in p‐MET high or p‐MET low tumors. ^*^ *p* < 0.05. J,K) Quantification analysis of GzmB^+^CD8^+^ T cells (J) and Perforin^+^CD8^+^ T cells (K) in p‐MET^high^ and p‐MET^low^ tumors. n = 5 per group. ^*^ *p* < 0.05. L) Quantification analysis of Ki67^+^ CD8^+^ T cells infiltration in p‐MET^high^ and p‐MET^low^ tumors. *n* = 5 per group. ^*^ *p* < 0.05.

A recent study by a U.S.‐based research group reported a positive association between tumor‐intrinsic PD‐1 expression and MET phosphorylation.^[^ ^11^ ^]^ To validate this relationship, we performed IHC staining for p‐MET on our PDAC TMA. High p‐MET expression was observed in 13 of 29 patients (44.8%), and PD‐1 expression positively correlated with p‐MET levels (p = 0.048, **Figure** **1**A,B; Figure S1E and Table S1, Supporting Information). To evaluate MET pathway activity, we performed GSVA using the BIOCARTA_MET_PATHWAY gene set from the MSigDB database. Consistent with IHC results, PD‐1 expression was significantly positively correlated with MET pathway activation scores in the TCGA cohort (p<0.001, Figure S1F, Supporting Information). Collectively, these findings reinforce that tumor‐intrinsic PD‐1 is both a prognostic biomarker and a molecular correlate of MET signaling activation in PDAC.

### 2.2 Activation of MET is Linked to a Poor Prognosis and an Immunosuppressive Contexture in PDAC

MET is normally expressed in epithelial cells and plays a pivotal role in various physiological processes. However, in cancer, MET is frequently overexpressed and contributes to tumor progression by activating downstream oncogenic signaling pathways.^[^ ^18^ ^]^ To assess MET activation in PDAC, we analyzed two publicly available transcriptomic datasets and observed significantly higher MET pathway activity in PDAC samples compared to normal pancreatic tissues (*p* < 0.001, Figure S2A, Supporting Information). IF staining further confirmed that p‐MET was predominantly localized in malignant epithelial cells (Figure S2B, Supporting Information). To distinguish MET expression from its activation status, we performed IHC for total MET in our 29‐case PDAC TMA. Interestingly, elevated total MET expression did not always correlate with high p‐MET levels in individual tumor specimens (Figure S2C, Supporting Information), and no significant correlation was found between tumor‐intrinsic PD‐1 and total MET expression (Figure S2D, Supporting Information). These findings support the conclusion that total MET abundance does not reliably indicate pathway activation, and p‐MET serves as a more definitive biomarker of MET signaling in PDAC.

Clinically, high p‐MET expression was associated with significantly worse OS (21.1 months vs. N.A., p = 0.017, HR = 3.258, Figure 1C) and shorter RFS (8.0 months vs 29.2 months, p = 0.003, HR = 3.497, Figure 1D). Then, multivariate analysis confirmed that p‐MET was an independent prognostic factor for both OS (p = 0.025, HR = 3.604, 95% CI: 1.173‐11.075) and RFS (p = 0.031, HR = 3.032, 95% CI: 1.109‐8.291) (Table S2, Supporting Information). Consistent results were obtained from the TCGA cohort, where patients with MET pathway activation also had inferior OS (19.8 months vs. 24.6 months, p = 0.013, HR = 1.708, 95% CI: 1.138‐2.576, Figure S2E, Supporting Information) and RFS (13.8 months vs. 17.5 months, p = 0.048, HR = 1.478, 95% CI: 1.005‐2.174, Figure S2F, Supporting Information).

In various cancers, MET activation has been linked to tumor progression, metastasis, and the development of an immunosuppressive TME.^[^ ^19^ ^]^ However, its specific role in shaping immune dynamics within the PDAC microenvironment remains incompletely understood. Among tumor‐infiltrating immune cells, CD8⁺ T cells play a pivotal role in antitumor immunity and are widely regarded as indicators of effective immune responses. To investigate the immunological consequences of MET activation in PDAC, we re‐analyzed the relationship between p‐MET expression and the functional states of tumor‐infiltrating CD8⁺ T cells (activation and exhaustion).^[^ ^20^ ^]^ Intriguingly, flow cytometry analysis revealed that p‐MET‐high tumors exhibited significantly reduced cytotoxic function, as indicated by decreased expression of GzmB (p = 0.009) and perforin (*p* < 0.001) (Figure 1E; Figure S3A, Supporting Information). Concurrently, these tumors showed increased expression of exhaustion markers, including PD‐1 (p = 0.001), TIM‐3 (*p* < 0.001), and TIGIT (*p* < 0.001) (Figure 1F; Figure S3B, Supporting Information). No obvious differences were found in CTLA‐4, TNF‐a or IFN‐γ expression in CD8^+^ T cells (Figure S3C–E, Supporting Information). These findings were corroborated by mIF which demonstrated that p‐MET‐high PDAC tumors were enriched in TIM‐3⁺PD‐1⁺ exhausted CD8⁺ T cell subsets (p = 0.020, Figure 1G,H), and had markedly lower frequencies of GzmB⁺ and Perforin⁺ effector cells (Figure 1I–K). Furthermore, the proportion of proliferating Ki67⁺ CD8⁺ T cells was also significantly reduced in p‐MET‐high tumors (p = 0.035, Figure 1L; Figure S4A, Supporting Information). Finally, applying validated gene signatures of pro‐tumoral immune contexture,^[^ ^21^ ^]^ we found that MET pathway activation was associated with significant enrichment of immunosuppressive TME features in the TCGA cohort (p = 0.001, Figure S4B, Supporting Information). In summary, these data identify MET activation as not only a prognostic marker for poor clinical outcomes but also as a key contributor to the development of an immunosuppressive microenvironment in PDAC through the functional exhaustion of tumor‐infiltrating CD8⁺ T cells.

### 2.3 Activation of MET Promotes the Accumulation of GITR^+^ Tregs in PDAC

**Figure 2:**
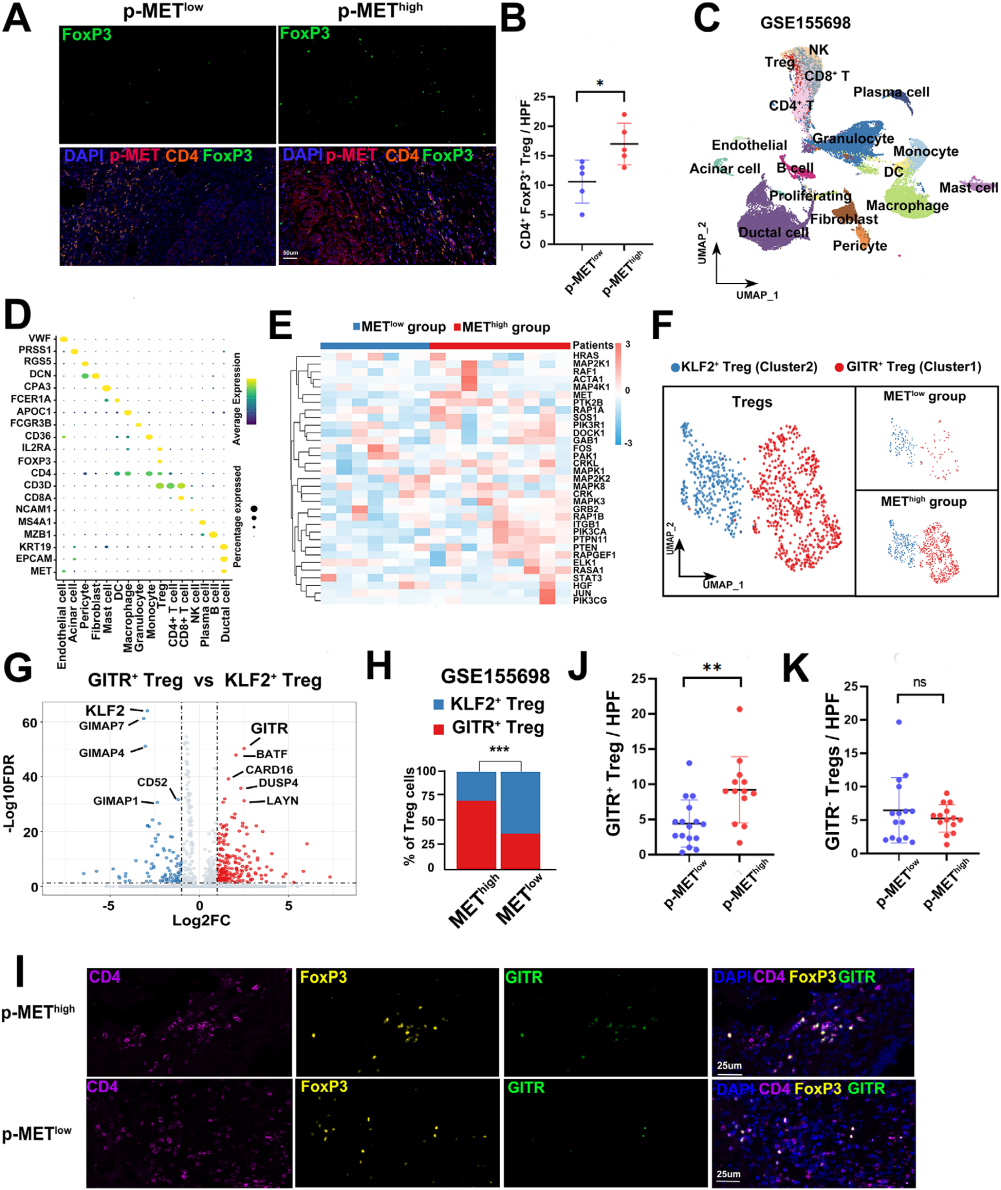
MET activation is associated with GITR^+^ Tregs accumulation in PDAC. A) Representative immunofluorescence staining images for tumor‐infiltrating Tregs in p‐MET high or p‐MET low PDAC tissues. B) Quantification analysis of Tregs infiltration in p‐MET^high^ or p‐MET^low^ PDAC patients. *n* = 5 per group. ^*^ *p* < 0.05. C) UMAP plots of single cells identified by scRNA‐seq and colored by major cell types. D) Dotplot displaying marker genes of different cell types. E) Heatmap exhibiting MET pathway related genes expression in activation score of MET^high^ and MET^low^ group. F) UMAP plots of Tregs in MET^high^ and MET^low^ group colored by different Tregs subclusters. (G) Differentially expressed genes between GITR^+^ Tregs and KLF2^+^ Tregs. H) Percentages of GITR^+^ Tregs and KLF2^+^ Tregs in MET^high^ or MET^low^ group from GSE155698 datasets. ^***^ *p* < 0.001. I) Representative immunofluorescence staining images for tumor‐infiltrating GITR^+^ Tregs and GITR^−^ Tregs in p‐MET high or p‐MET low tumors. J,K) Quantification of GITR^+^ Tregs (J) and GITR^−^ Tregs (K) infiltration in p‐MET^high^ and p‐MET^low^ PDAC patients. ^**^ *p* < 0.01. UMAP, uniform manifold approximation and projection; PDAC, pancreatic ductal adenocarcinoma.

To investigate the relationship between MET activation and immune components in PDAC, we re‐analyzed immune infiltration data from our previously published cohort of 29 cases.^[^ ^22^ ^]^ We examined the correlation between p‐MET expression and various immune cell subtypes within PDAC. The analysis revealed that only Tregs were significantly accumulated in PDAC patients with high MET activation, while no significant changes were observed in other immune cell types (Figure S5A; Table S1, Supporting Information). This association was validated in a second independent cohort (*n* = 10), where p‐MET‐high tumors exhibited significantly increased Treg infiltration (p = 0.022, **Figure** **2**A,B). To further confirm this finding, we applied the xCell algorithm to two independent bulk transcriptomic datasets (TCGA and E‐MTAB‐6134). In both cohorts, MET activation was positively correlated with Treg infiltration (Figure S5B, Supporting Information). These observations suggest that MET activation is positively correlated with the accumulation of Tregs, implicating it as a potential driver of immunosuppression in the PDAC TME.

While Tregs are known suppressors of anti‐tumor immunity in PDAC, they comprise a heterogeneous population with distinct functional subtypes.^[^ ^23^ ^]^ For instance, in ovarian cancer, effector Tregs (eTregs) were shown to mediate the therapeutic response to PARP inhibition,^[^ ^24^ ^]^ while in lung cancer, OX^hi^GITR^hi^ Treg were associated with resistance to PD‐1 blockade.^[^ ^25^ ^]^ Based on these insights, we focused on Treg subclusters rather than total Treg abundance.

We next examined the impact of MET activation on Treg subpopulations using the scRNA‐seq dataset GSE155698, which includes single‐cell transcriptomic data from 16 treatment‐naïve PDAC patients (totaling 42,941 cells). Using well‐established markers, these cells were annotated into 16 distinct clusters, including Tregs characterized by high expression of CD4, CD25, and FoxP3 (Figure 2C,D). Batch effects across samples were effectively minimized (Figure S5C, Supporting Information), and inferCNV analysis confirmed that all ductal epithelial cells were of malignant origin (Figure S5D, Supporting Information). Consistent with our mIF findings, MET expression was predominantly localized to malignant epithelial cells (Figure 2D). Using GSVA‐derived MET activation scores in ductal epithelial cells, patients were stratified into MET^high^ and MET^low^ groups. Nine patients (56.2%) were classified as MET^high^ (Figure 2E). As expected, both MET activation and MET transcript levels were significantly higher in the MET^high^ group (*p* < 0.001 and p = 0.016, respectively, Figure S5E,F, Supporting Information).

Analysis revealed a marked enrichment of Tregs in the MET^high^ group, accounting for 81.4% of total Tregs (Figure 2F). To investigate how MET activation influences Treg subclusters, we performed unsupervised clustering algorithms to further annotate the Tregs subclusters. This analysis identified two transcriptionally distinct subclusters: KLF2⁺ Tregs (Cluster 1), characterized by high KLF2 expression, a transcription factor associated with T cell naivety and regulation of CD8⁺ T cell exhaustion.^[^ ^26^ ^]^ GITR⁺ Tregs (Cluster 2), are defined by elevated GITR, a co‐stimulatory molecule implicated in immunoregulation and Treg activation.^[^ ^27^ ^]^ Based on prior studies associating KLF2 with resting Tregs and GITR with activated Tregs,^[^ ^24^ ^]^ we annotated these clusters as KLF2⁺ Tregs and GITR⁺ Tregs, respectively (Figure 2F,G). This classification was validated in two additional scRNA‐seq PDAC datasets, which consistently showed the same transcriptional distinction between KLF2 and GITR subclusters (Figure S5G,H, Supporting Information).

Notably, the increase in Tregs observed in MET^high^ group was predominantly attributed to the expansion of GITR^+^ Tregs, rather than KLF2^+^ Tregs (Figure 2F). A significantly higher proportion of GITR^+^ Tregs was observed in MET^high^ samples compared to MET^low^ (*p* < 0.001, Figure 2H). This trend was validated in the GSE263733 scRNA‐seq dataset, where GITR⁺ Tregs were significantly enriched in PDAC samples with high MET expression (*p* < 0.001, Figure S5I, Supporting Information). Furthermore, enrichment analysis using the GITR⁺ Treg gene signature in the TCGA, E‐MTAB‐6134, and ICGC cohorts confirmed a positive correlation between GITR^+^ Tregs and MET activation (Figure S5J, Supporting Information).

To further validate these findings, we performed mIF staining for CD4, FoxP3, and GITR on our 29‐case PDAC TMA. This further confirmed the presence of GITR^+^ Tregs (CD4^+^FoxP3^+^GITR^+^) in PDAC tissues (Figure 2I). Consistent with the scRNA‐seq results, patients with high p‐MET expression exhibited significantly higher infiltration of GITR^+^ Tregs (p = 0.003, Figure 2J). In contrast, the infiltration of GITR^−^ Tregs (CD4^+^FoxP3^+^GITR^−^) did not differ significantly (p = 0.403, Figure 2K). Taken together, these findings demonstrate that MET activation positively correlated with the infiltration of GITR^+^ Tregs in PDAC, highlighting a potential mechanism by which tumor‐intrinsic MET signaling may drive immune evasion through Treg reprogramming.

### 2.4 Increased Accumulation of GITR^+^ Tregs Correlates with Poorer Prognosis in PDAC

To improve the robustness of our prognostic analysis, we expanded our original cohort by including an additional 116 PDAC patients, resulting in a combined 145‐patient TMA cohort. Among these patients, 62.8% were male, and the median age was 64.9 years. Tumor differentiation was categorized as well‐differentiated in 6 patients (4.1%), moderately differentiated in 76 patients (52.4%), and poorly differentiated in 63 patients (43.4%). According to the AJCC 8^th^ TNM staging system, 71 patients (49.0%) were stage I, 64 patients (44.1%) stage II, and 10 patients (6.9%) stage III. The median preoperative serum levels of CA19‐9 and CEA were 80.6 (IQR: 19.5‐356.9) U mL^−1^ and 3.2 (IQR: 2.2‐5.3) ng mL^−1^, respectively. Detailed clinicopathological characteristics are provided in Table S3 (Supporting Information).

**Figure 3:**
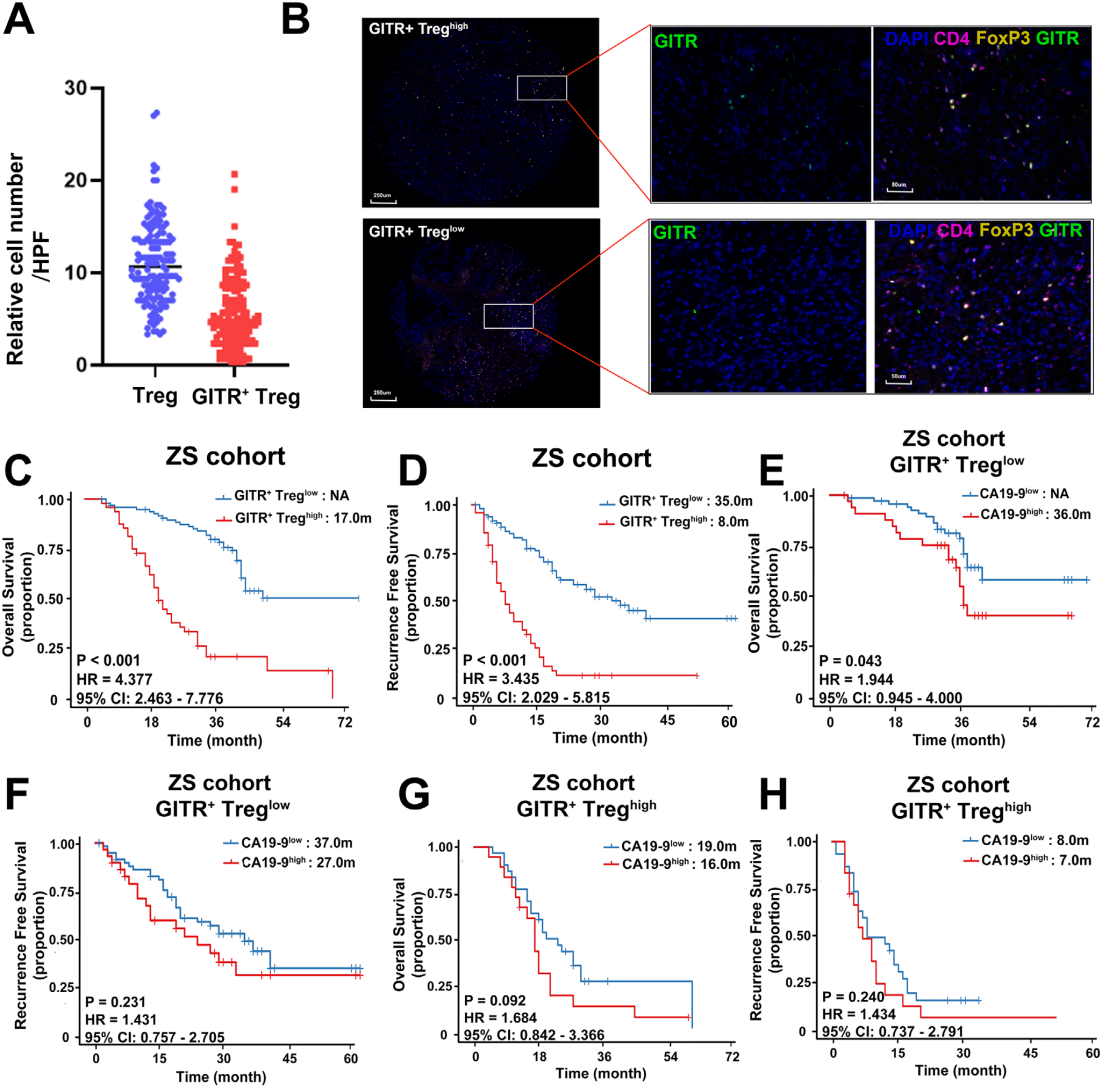
Tumor‐infiltrating GITR^+^ Tregs is correlated with poor survival in PDAC patients. A) Quantification of tumor‐infiltrating Tregs and GITR^+^ Tregs in ZS cohort (*n* = 145). B) Representative immunofluorescence images of high or low GITR^+^ Treg infiltration. C,D) Kaplan‐Meier curves for OS (C) and RFS (D) in PDAC patients with high or low GITR^+^ Tregs infiltrations in ZS cohort (*n* = 145). E,F) Kaplan‐Meier curves for OS (E) and RFS (F) in PDAC patients from ZS cohort stratifying by serum CA19‐9 in low GITR^+^ Tregs infiltration groups. G,H) Kaplan‐Meier curves for OS and RFS in PDAC patients from ZS cohort stratifying by serum CA19‐9 in high GITR^+^ Tregs infiltration group. OS, overall survival; RFS, recurrence‐free survival; PDAC, pancreatic ductal adenocarcinoma.

The presence of GITR^+^ Tregs within the PDAC microenvironment was assessed using IF staining. The median infiltration densities of GITR^+^ Tregs and total conventional Tregs were 4.7 (IQR: 2.7‐8.3) /HPF and 10.6 (IQR: 7.7‐13.8) /HPF in the TMA slices, respectively (**Figure** **3**A; Table S3, Supporting Information). High infiltration of GITR^+^ Tregs was observed in 33.1% of PDAC patients. Representative images of GITR^+^ Treg infiltration in both groups are shown in Figure 3B. Clinically, patients with elevated levels of GITR^+^ Tregs had a higher risk of lymph node metastasis (**Table** **1**). Kaplan‐Meier survival analysis demonstrated that high infiltration of GITR^+^ Tregs was significantly associated with reduced OS (17.0 months vs. N.A., p < 0.001, HR = 4.377, 95% CI: 2.463‐7.776, Figure 3C) and RFS (8.0 months vs. 35.0 months, p < 0.001, HR = 3.435, 95% CI: 2.029‐5.815, Figure 3D). Similarly, higher levels of total conventional Tregs were also associated with worse OS (20.0 months vs. 37.0 months, p < 0.001, HR = 2.600, 95% CI: 1.520‐4.445, Figure S6A, Supporting Information) and RFS (10.0 months vs. 29.0 months, p < 0.001, HR = 2.121, 95% CI:1.297‐3.469, Figure S6B, Supporting Information), but to a lesser extent. Receiver operating characteristic (ROC) curve analysis demonstrated the superior prognostic accuracy of GITR⁺ Treg infiltration for OS at 1, 3, and 5 years (AUC = 0.820, 0.798, and 0.839, respectively), compared to total Tregs or GITR⁻ Tregs (Figure S6C, Supporting Information). Similar results were observed for RFS prediction, with AUCs of 0.808, 0.782, and 0.922, respectively (Figure S6D, Supporting Information). Interestingly, higher GITR⁻ Treg infiltration was associated with longer OS (57.0 months vs. 34.0 months, p = 0.016, HR = 0.533, 95% CI: 0.332‐0.855, Figure S6E, Supporting Information) and showed a trend toward improved RFS (29.0 months vs. 17.0 months, p = 0.073, HR = 0.661, 95% CI: 0.428‐1.021, Figure S6F, Supporting Information). These results suggest that GITR⁻ Tregs may represent a less immunosuppressive or even protective phenotype, potentially reflecting an active immune response. These findings collectively demonstrate that GITR stratifies tumor‐infiltrating Tregs into two prognostically distinct populations, which highlights the importance of Treg heterogeneity in understanding and predicting patient prognosis in PDAC.

**Table 1:** The relationships between tumor‐infiltrating Tregs and GITR^+^ Tregs and clinicopathological characteristics.

Variables	Low Tregs	High Tregs	*P* value	Low GITR^+^ Tregs	High GITR^+^ Tregs	*P* value
	(*n* = 96)	(*n* = 49)		(*n* = 97)	(*n* = 48)	
**Sex**			0.173			0.254
Male/ Female	64/32	27/22		64/33	27/21	
**Age (years)**			0.730			0.857
<65/ ≥ 65	46/50	22/27		46/51	22/26	
**Differentiation**			0.266			0.448
I/ II/ III	5/54/37	1/22/26		5/53/39	1/23/24	
**T stage**			0.615			0.051
T1/T2/ T3	33/49/14	13/29/7		37/46/14	9/32/7	
**N stage**			0.332			**0.033**
N0/N1/N2	59/31/6	24/21/4		62/31/4	21/21/6	
**TNM stage** I/ II/ III	51/39/6	20/25/4	0.356	52/41/4	19/23/6	0.092
**CA19‐9**			0.684			0.591
<200/≥200 U mL^−1^	64/32	31/18		65/32	30/18	
**CEA**			0.666			0.582
<5/≥5 ng mL^−1^	68/28	33/16		69/28	32/16	
**ART**			0.076			0.376
No / Yes	65/31	40/9		68/29	37/11	
**ACT**			0.740			0.692
No / Yes	10/86	6/43		10/87	6/42	
**Microvascular invasion**			0.403			0.083
No / Yes	71/25	33/16		74/23	30/18	
**Perineural invasion**			0.233			0.106
No / Yes	17/79	5/44		18/79	4/44	
**Tumor GITR^+^ Tregs** Low/ High	89/7	8/41	**<0.001**	/	/	/
**Tumor Tregs** Low/ High	/	/	/	89/8	7/41	**<0.001**

After the univariate and multivariate analysis, T stage (p = 0.005, HR = 1.722, 95% CI: 1.183‐2.505), ACT (p < 0.001, HR = 0.292, 95% CI: 0.142‐0.604), and GITR^+^ Treg (*p* < 0.001, HR = 5.822, 95% CI: 2.656‐12.761) were identified as independent prognostic factors for OS. Notably, only T stage (*p* < 0.001, HR = 1.819, 95% CI: 1.277‐2.591) and GITR^+^ Treg (*p* < 0.001, HR = 4.122, 95% CI: 2.085‐8.149) remained significant independent prognostic factors for RFS (**Table** **2**). To externally validate these findings, we applied the GITR⁺ Treg gene signature to the TCGA cohort, stratifying patients into high vs. low GITR⁺ Treg groups. Consistent with our clinical cohort, patients with high GITR⁺ Treg scores had significantly worse OS (19.8 months vs. 23.4 months, p = 0.021, HR = 1.770, 95% CI:1.148‐2.730, Figure S6G, Supporting Information) and RFS (14.2 months vs. 19.9 months, p = 0.010, HR = 1.797, 95% CI: 1.197‐2.697, Figure S6H, Supporting Information).

**Table 2:** Univariate and multivariate analysis of prognostic indicators associated with overall survival and recurrence‐free survival.

Variables	Overall survival			Recurrence‐free survival		
	Univariate *P* value	Multivariate *P* value	Multivariate HR (95% CI)	Univariate *P* value	Multivariate *P* value	Multivariate HR (95% CI)
**Sex**						
Male/Female	0.844	NA		0.659		
**Age (years)**						
<65/≥65	0.531	NA		0.832		
**Differentiation**						
I/II/III	**0.035**	0.201	1.364 (0.847‐2.196)	0.171		
**T stage**						
T1/T2/ T3	**0.014**	**0.005**	1.722 (1.183‐2.505)	**0.003**	**<0.001**	1.819 (1.277‐2.591)
**N stage**						
N0/N1/N2	**<0.001**	0.351	1.194 (0.822‐1.735)	**0.023**	0.212	1.246 (0.882‐1.759)
**CA19‐9**						
<200/≥200 U mL^−1^	**0.020**	0.187	1.420 (0.843‐2.390)	0.094		
**CEA**						
<5/≥5 ng mL^−1^	0.155	NA		0.167		
**ART**						
No / Yes	0.969	NA		0.194		
**ACT**						
No / Yes	**0.022**	**<0.001**	0.292 (0.142‐0.604)	0.566		
**Microvascular invasion**						
No / Yes	0.094	NA		0.225		
**Perineural invasion**						
No / Yes	**0.024**	0.259	1.786 (0.652‐4.888)	**0.029**	0.063	2.052 (0.962‐4.378)
**Tumor GITR^+^ Tregs** Low/ High	**<0.001**	**<0.001**	5.822 (2.656‐12.761)	**<0.001**	**<0.001**	4.122 (2.085‐8.149)
**Tumor Tregs** Low/ High	**<0.001**	0.305	0.683 (0.329‐1.415)	**<0.001**	0.381	0.750 (0.393‐1.429)

### 2.5 Prognostic Variations of Serum CA19‐9 Levels are Modified by Tumor‐Infiltrating GIRT^+^ Tregs

CA19‐9 is the most widely used serum biomarker for PDAC, approved by the U.S. Food and Drug Administration for diagnosis, monitoring tumor burden, and evaluating treatment response. Its preoperative and perioperative prognostic value has been consistently demonstrated across multiple clinical studies.^[^ ^28^ ^]^ To explore whether the prognostic utility of CA19‐9 is influenced by the TIME, we conducted an interaction analysis stratified by levels of tumor‐infiltrating GITR⁺ Tregs after adjusting for potential confounders, the analysis revealed a significant interaction between GITR⁺ Treg infiltration and the prognostic effect of CA19‐9 for both OS and RFS (*p* < 0.001 for both).

Among patients with low GITR^+^ Treg infiltration, elevated CA19‐9 levels retained strong prognostic significance. Specifically, patients with high CA19‐9 had significantly shorter median OS compared to those with low CA19‐9 (36 months vs. N.A., p = 0.043, HR = 1.944, 95% CI: 0.945‐4.000, Figure 3E), and showed a trend toward shorter RFS (27 months vs. 37 months, p = 0.231, HR = 1.431, 95% CI: 0.757‐2.705, Figure 3F). In contrast, among patients with high GITR⁺ Treg infiltration, CA19‐9 levels failed to significantly stratify patient outcomes. OS was comparable between high and low CA19‐9 subgroups (16 months vs. 19 months, p = 0.092, HR = 1.684, 95% CI: 0.842‐3.366, Figure 3G), as was RFS (7 months vs. 8 months, p = 0.240, HR = 1.434, 95% CI: 0.737‐2.791, Figure 3H). These findings suggest that the immunosuppressive TME, shaped by abundant GITR^+^ Tregs, may attenuate the prognostic relevance of CA19‐9. In other words, in highly immunosuppressive tumors, immune‐mediated mechanisms may override the tumor‐intrinsic biological signals typically captured by CA19‐9 levels. This underscores the importance of the TME context in interpreting biomarker performance and highlights the potential need for integrating immune profiling with serum biomarker data to improve prognostic accuracy in PDAC.

### 2.6 GITR^+^ Tregs Represent a Distinct Effector Subset with Intensive Immunosuppressive Function

**Figure 4:**
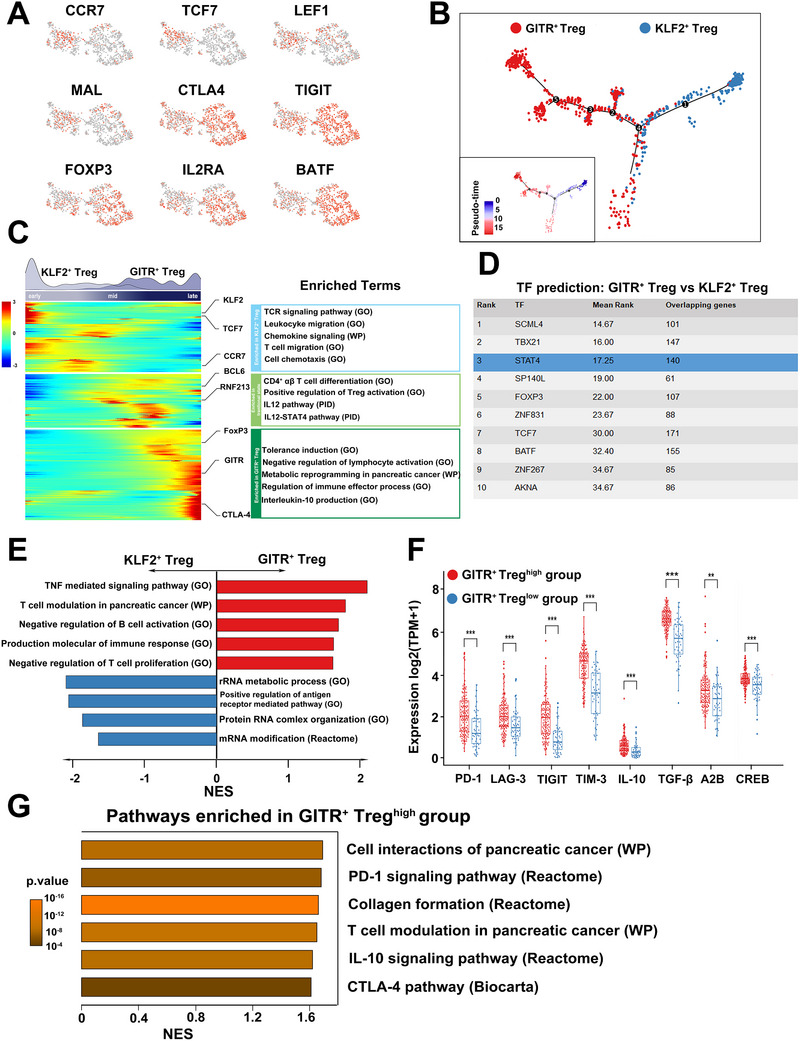
GITR^+^ Tregs represent a specific subset with intensive immunosuppressive effector function. A) UMAP plots displaying indicated immune genes expression in tumor infiltrating Tregs in GSE155698 dataset. B) Trajectory manifold of KLF2^+^ Tregs and GITR^+^ Tregs using monocle2 algorithm. (C) Heatmap exhibiting the dynamic changes and enriched pathways of highly variable genes across pseudo‐time in Tregs. D) Transcription factor predicted to be activated in GITR^+^ Tregs or KLF2^+^ Tregs. E) Gene Set Enrichment Analysis for genes ranked by log2 fold change between GITR^+^ Tregs and KLF2^+^ Tregs. F) Immune genes expression between high and low GITR^+^ Tregs groups in PDAC patients from TCGA cohort. G) Pathways enriched in high GITR^+^ Tregs group in patients from TCGA cohort. TCGA, The Cancer Genome Atlas; PDAC, pancreatic ductal adenocarcinoma; UMAP, uniform manifold approximation and projection.

Our findings demonstrate that GITR⁺ Tregs, unlike total Tregs, serve as an independent prognostic factor in PDAC, suggesting they represent a functionally distinct and more immunosuppressive subset. Transcriptomic profiling revealed that KLF2⁺ Tregs exhibited high expression of resting Treg‐associated genes, including CCR7, TCF7, and LEF1, whereas GITR⁺ Tregs showed marked upregulation of effector‐associated markers such as CTLA4, TIGIT, and FoxP3 (**Figure** **4**A). These molecular signatures suggest that KLF2⁺ Tregs represent a resting or naïve subset, while GITR⁺ Tregs constitute an activated effector population. To explore the developmental trajectory of these subpopulations, we performed pseudo‐time analysis, which positioned KLF2⁺ Tregs at the early stages and GITR⁺ Tregs at the terminal end of the differentiation continuum (Figure 4B). This progression suggests that KLF2^+^ Tregs may serve as precursors that differentiate into GITR⁺ effector Tregs upon activation.

Within the intermediate trajectory state, we observed upregulation of differentiation‐associated genes, including RNF213 and BCL6, accompanied by enrichment of pathways related to CD4⁺ αβ T cell differentiation and T cell activation. These findings support a model in which resting KLF2⁺ Tregs transition into GITR⁺ Tregs under activating stimuli. Interestingly, STAT4 signaling emerged as a key pathway enriched during this transitional state (Figure 4C). Computational inference of transcription factor activity identified STAT4 as a critical regulator of 140 DEGs between KLF2^+^ and GITR^+^ Tregs, including hallmark effector genes such as GITR, TNFRSF4, TIGIT, and CTLA‐4 (Figure 4D). Notably, STAT4 expression peaked during the intermediate state of the differentiation trajectory—later than canonical Treg transcription factors such as FoxP3 and BATF, which were upregulated early and remained elevated in terminally differentiated cells (Figure S7A, Supporting Information). These results suggest that STAT4 plays a pivotal role in orchestrating the transition from resting to effector Tregs.

Gene enrichment and GSEA revealed that GITR^+^ Tregs are significantly associated with immunoregulatory pathways, including those involved in T cell modulation, negative regulation of T cell proliferation, and metabolic reprogramming linked to PDAC. In contrast, KLF2^+^ Tregs are primarily enriched in pathways related to rRNA metabolism, antigen receptor mediated signaling pathway and chemotaxis (Figure 4C,E). These findings suggest that GITR^+^ Tregs may possess a stronger immunoregulatory capacity than KLF2^+^ Tregs. Furthermore, Tregs mediate immunosuppression through four major mechanisms: inhibitory cytokines, metabolic disruption, cytolysis, and directly targeting antigen‐presenting cells (APCs).^[^ ^29^, ^30^ ^]^ Applying established gene signatures, we found that GITR^+^ Tregs more strongly relied on metabolic disruption (*p* < 0.001) and targeting APC (*p* < 0.001) than KLF2^+^ Tregs (Figure S7B, Supporting Information). Specifically, CD39 and CTLA‐4, key mediators of adenosine generation and APC suppression, respectively, were significantly upregulated in GITR⁺ Tregs (Figure S7C, Supporting Information). These findings were validated in an independent PDAC scRNA‐seq dataset (GSE263733; Figure S8A–D, Supporting Information). Together, these observations indicate that GITR^+^ Tregs from an effector subset of Tregs with substantially greater immunosuppressive capacity than KLF2^+^ Tregs. This enhanced functionality is largely driven by their ability to disrupt metabolic pathways and inhibit APC activity within the PDAC microenvironment.

To functionally validate the impact of GITR⁺ Tregs on the tumor microenvironment, we compared immune profiles between computationally predicted high versus. low GITR⁺ Treg‐infiltrated PDAC tumors using TCGA data. The high GITR⁺ Treg group displayed elevated levels of exhaustion markers (PD‐1, LAG‐3, TIM‐3, TIGIT), and inhibitory cytokines (IL‐10, TGF‐β), as well as metabolic suppressors (CREB, adenosine receptor A2B) (Figure 4F). GSEA further revealed that this group was enriched in CTLA‐4 and PD‐1 signaling pathways as well as extracellular matrix (ECM)‐related remodeling programs (Figure 4G), all of which are known to contribute to immune evasion in PDAC. These findings confirm that a GITR^+^ Treg‐enriched TME is more immunosuppressive, contributing to impaired anti‐tumor immunity in PDAC.

### 2.7 MET Activation Drives GITR^+^ Tregs Accumulation and Immunosuppression

**Figure 5:**
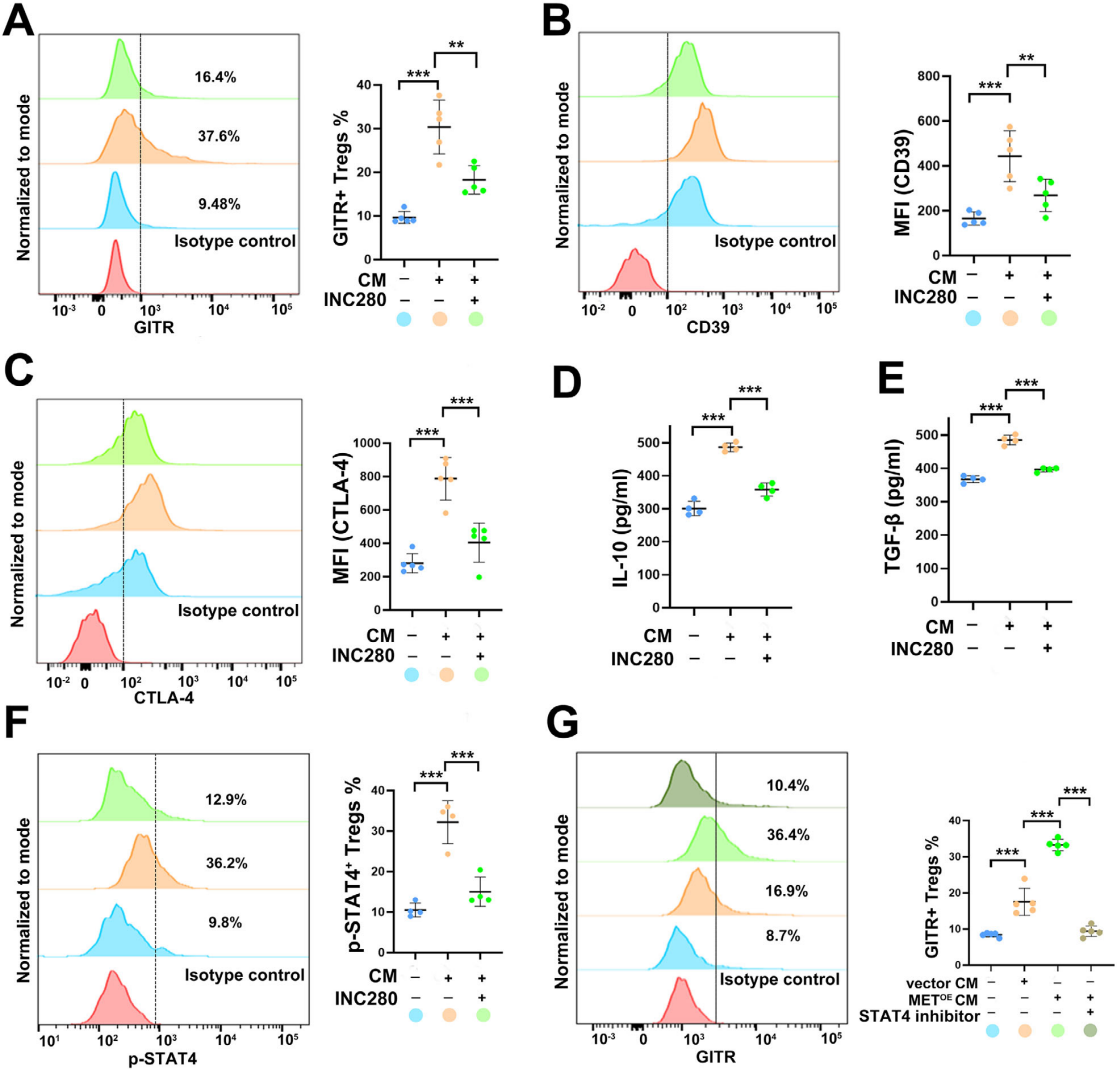
PDAC cells promote GITR^+^ Tregs differentiation and enhance immunosuppressive capacity via tumor‐intrinsic MET activation A) Flow cytometry analysis of GITR^+^ Treg proportion with/without CFPAC‐1 CM treatment in the presence or absence of INC280. *n* = 5 per group. ^**^ *p* < 0.01, ^***^ *p* < 0.001. B,C) Flow cytometry analysis of CD39 B) and CTLA‐4 C) expression in Tregs with/without CFPAC‐1 CM treatment in the presence or absence of INC280. *n*=5 per group. ^**^ *p *< 0.01, ^***^ *p* < 0.001. (D, E) ELISA assay for supernatant IL‐10 D) and TGF‐β E) levels from Tregs with/without CFPAC‐1 CM treatment in the presence or absence of INC280. *n*=4 per group. ^***^ *p* < 0.001. F) Flow cytometry analysis of pSTAT4 expression in Tregs with/without CFPAC‐1 CM treatment in the presence or absence of INC280. *n*=4 per group. ^***^ *p* < 0.001. G) Flow cytometry analysis of GITR^+^ Tregs proportion with/without MIA‐PaCa‐2 vector /MET^OE^ CM treatment in the presence or absence of STAT4 inhibitor. *n*=5 per group. ^***^ *p *< 0.001. CM, conditioned medium; ELISA, Enzyme‐Linked Immunosorbent Assay.

To investigate the TME‐driven mechanisms underlying GITR^+^ Treg accumulation, we performed in vitro functional assays using CM derived from human PDAC cell lines. Human Tregs were induced from naïve CD4⁺ T cells, with purity confirmed by FoxP3 expression via flow cytometry (Figure S9A, Supporting Information).

p‐MET expression was elevated in multiple human PDAC cell lines, including AsPC‐1, CFPAC‐1 and BxPC‐3 (Figure S9B, Supporting Information). The selective MET inhibitor INC280 was used to suppress p‐MET activity, and its efficacy was validated by western blot analysis (Figure S9C, Supporting Information). To model the influence of PDAC‐derived soluble factors on Tregs, we stimulated Tregs with CM from CFPAC‐1 cells. Flow cytometry analysis revealed that GITR^+^ Tregs comprised ≈10% of the Treg population in untreated conditions, whereas stimulation with CFPAC‐1 CM significantly increased GITR⁺ Treg frequency (p < 0.001). This effect was abrogated when the CM was derived from CFPAC‐1 cells pretreated with INC280 (p = 0.001), suggesting that MET activation is required to drive GITR⁺ Treg differentiation (**Figure** **5**A). A similar trend was observed using CM from BxPC‐3 cells (Figure S9D, Supporting Information).

To evaluate whether MET activation also enhances the immunosuppressive function of Tregs, we assessed the expression of canonical inhibitory molecules and immunoregulatory cytokines.^[^ ^30^ ^]^ Notably, CD39 and CTLA‐4—key effectors of Treg‐mediated suppression—were significantly upregulated upon CFPAC‐1 CM treatment (p < 0.001 for both), and were significantly reduced by INC280 pretreatment (CD39: p = 0.009, CTLA‐4: *p* < 0.001, Figure 5B,C). Consistently, ELISA analysis showed that stimulation with CFPAC‐1 CM led to enhanced secretion of IL‐10 and TGF‐β, both hallmark immunosuppressive cytokines (*p* < 0.001 for both). These effects were reversed in the presence of MET inhibition (Figure 5D,E). Together, these findings demonstrate that MET activation in PDAC cells not only promotes the accumulation of GITR⁺ Tregs but also amplifies their immunosuppressive function through the upregulation of checkpoint molecules and immunoregulatory cytokines. This dual role highlights the therapeutic potential of targeting the MET–GITR⁺ Treg axis as a strategy to counteract immune suppression and restore antitumor immunity in PDAC.

### 2.8 MET Activation Reprograms GITR^+^ Tregs through IL‐23/STAT4 Signaling

Given bioinformatic evidence implicating STAT4 in the differentiation of GITR⁺ Tregs, we examined whether STAT4 phosphorylation mediates MET‐driven GITR⁺ Treg accumulation. Flow cytometry revealed a significant increase in p‐STAT4 levels in Tregs following stimulation with CM from CFPAC‐1 cells (*p* < 0.001, Figure 5F). This effect was markedly diminished when CM was derived from INC280‐pretreated cells, confirming that MET signaling enhances STAT4 phosphorylation (*p* < 0.001, Figure 5F). To assess the role of STAT4, we performed a rescue experiment using *p*‐biphenyl phosphate, a selective STAT4 inhibitor. In MET^OE^ MIA‐PaCa‐2 cells (Figure S9E, Supporting Information), CM significantly increased the proportion of GITR⁺ Tregs compared to CM from control cells (*p* < 0.001, Figure 5G). Notably, this effect was reversed by STAT4 inhibition (*p* < 0.001, Figure 5G), and p‐STAT4 levels mirrored this pattern (Figure S9F, Supporting Information), confirming STAT4 activation as a critical mediator of MET‐driven GITR⁺ Treg reprogramming.

**Figure 6:**
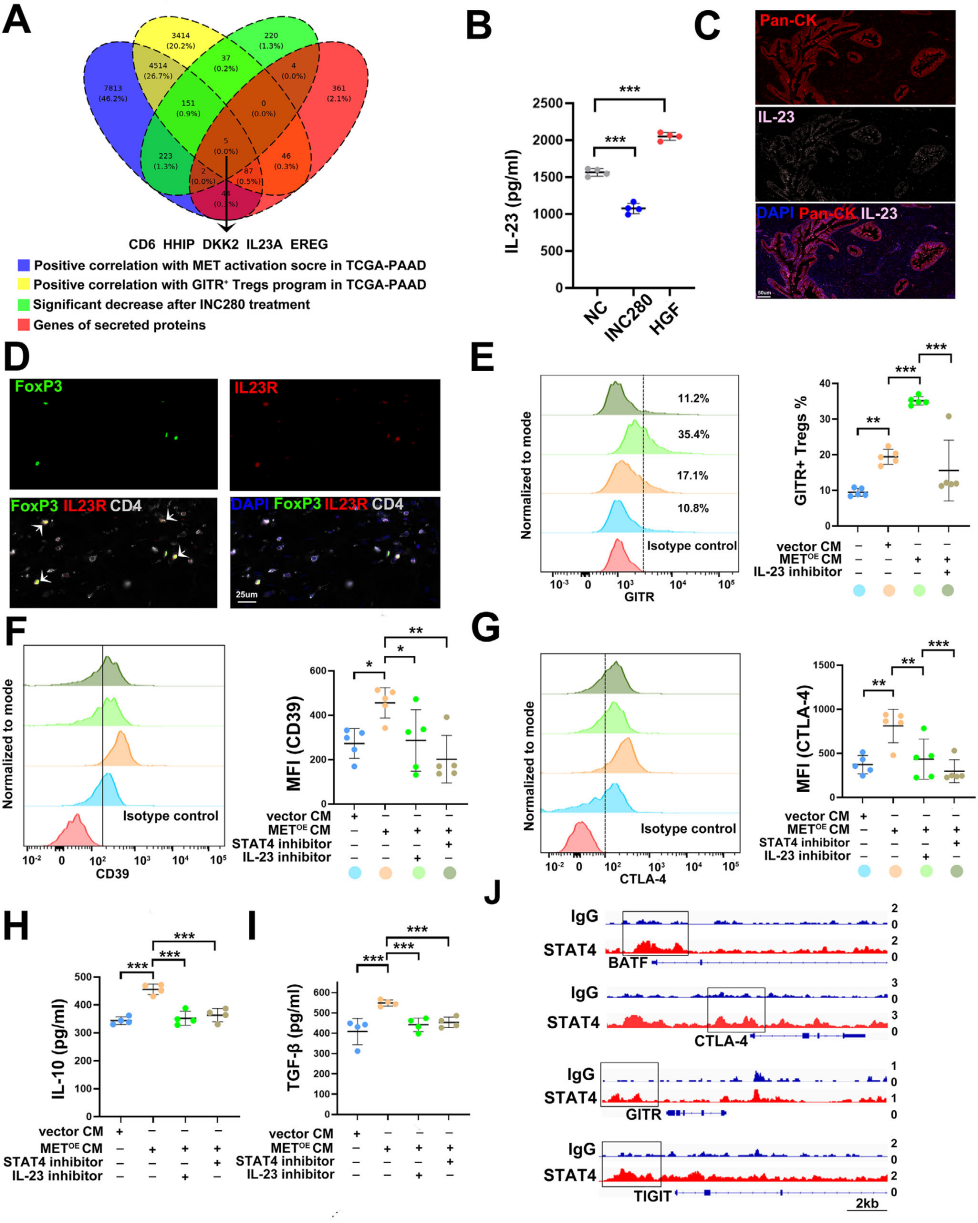
MET induces GITR^+^ Tregs accumulation through IL‐23/STAT4 signaling axis. A) Integrated analysis for secreted proteins that is promoted by MET activation and drives GITR^+^ Tregs accumulation. B) ELISA assay for supernatant IL‐23 levels from CFPAC‐1 with INC280 or HGF treatment. *n* = 5 per group. ^***^ *p* < 0.001. C) Representative immunofluorescence staining images for co‐localization of pan‐CK and IL‐23. D) Representative immunofluorescence staining images for co‐localization of IL‐23R and Tregs. E) Flow cytometry analysis of GITR^+^ Tregs proportion with/without MIA‐PaCa‐2 vector /MET^OE^ CM treatment in the presence or absence of IL‐23 inhibitor. *n* = 5 per group. ^**^ *p* < 0.01, ^***^ *p* < 0.001. F,G) Flow cytometry analysis of CD39 (F) and CTLA‐4 (G) expression on Tregs with/without MIA‐PaCa‐2 vector /MET^OE^ CM treatment in the presence or absence of IL‐23 inhibitor or STAT4 inhibitor. *n* = 5 per group. ^*^ *p* < 0.05, ^**^ *p* < 0.01, ^***^ *p* < 0.001. (H,I) ELISA assay for supernatant IL‐10 and TGF‐β levels from Tregs with/without MIA‐PaCa‐2 vector /MET^OE^ CM treatment in the presence or absence of IL‐23 inhibitor or STAT4 inhibitor. *n* = 4 per group. ^***^ *p* < 0.001. J) CUT&RUN analysis of STAT4 binding peaks around GITR^+^ Tregs associated genes. CM, conditioned medium; ELISA, Enzyme‐Linked Immunosorbent Assay; CUT&RUN, Cleavage under targets and release using nuclease.

To identify upstream mediators of this process, we performed bulk RNA sequencing on CFPAC‐1 cells treated with or without INC280. Candidates were selected based on four stringent criteria: 1) significant downregulation upon MET inhibition, 2) positive correlation with MET activation in TCGA, 3) positive correlation with the GITR^+^ Treg signature, and 4) classification as secreted proteins based on CellPhoneDB annotations. Five genes met these criteria (**Figure** **6**A), among which *IL‐23A* and *DKK2* were significantly reduced after MET inhibition (Figure S9G, Supporting Information). IL‐23, a cytokine in the IL‐12 family, is known to activate STAT4 and stabilize effector Tregs, whereas DKK2 is a Wnt signaling antagonist signaling through KREMEN, a receptor absent in Tregs (Figure S9H, Supporting Information).^[^ ^31^, ^32^ ^]^ Thus, IL‐23 was prioritized as the key functional link between MET and STAT4 in Tregs.

Then, ELISA analysis confirmed that IL‐23 secretion decreased following INC280 treatment and increased after HGF (a MET agonist) stimulation (*p* < 0.001, Figure 6B). The scRNA‐seq analysis and mIF staining further validated that IL‐23 is predominantly produced by tumor cells in PDAC (Figure 6C; Figure S9I, Supporting Information). Co‐immunostaining revealed IL‐23R expression on Tregs, supporting the existence of a direct IL‐23 signaling axis in GITR⁺ Treg differentiation (Figure 6D).

To assess the clinical relevance of this axis, we analyzed two independent PDAC transcriptomic cohorts (E‐MTAB‐6134 and GSE71729). Both datasets showed a positive correlation between *IL‐23A* expression and the GITR⁺ Tregs (Figure S9J, Supporting Information), further reinforcing IL‐23 as a functional driver of GITR⁺ Treg enrichment in PDAC. Collectively, these results establish IL‐23 as a critical mediator in the MET‐driven immunoregulatory pathway that promotes GITR⁺ Treg accumulation.

To further investigate whether MET promotes GITR^+^ Tregs accumulation through IL‐23/STAT4 axis, we performed a rescue experiment using the IL‐23‐neutralizing antibody tildrakizumab. CM from MET^OE^ MIA‐PaCa‐2 cells significantly increased the proportion of GITR⁺ Tregs compared to CM from control cells (p < 0.001), while IL‐23 blockade reversed this effect (*p* < 0.001, Figure 6E). Correspondingly, p‐STAT4 levels were elevated upon MET^OE^ CM stimulation and were markedly reduced by IL‐23 inhibition (Figure S9K, Supporting Information). These results, together with our previous STAT4 inhibition data, establish that MET promotes GITR⁺ Treg polarization through IL‐23–dependent STAT4 activation, identifying a targetable immunosuppressive axis in PDAC.

To further delineate the functional consequences of this signaling cascade, we examined the surface expression of key immunosuppressive markers and cytokine production in Tregs. Both STAT4 and IL‐23 inhibition significantly downregulated CD39 and CTLA‐4 expression (Figure 6F,G), while ELISA confirmed reduced secretion of IL‐10 and TGF‐β under the same treatment conditions (Figure 6H,I). These findings indicate that IL‐23–STAT4 signaling not only drives GITR⁺ Treg accumulation but also enhances their suppressive function.

Mechanistically, CUT&RUN data from human Tregs (GSE252765)^[^ ^33^ ^]^ demonstrated direct STAT4 binding to the regulatory regions of multiple GITR⁺ Treg‐associated genes, including CTLA‐4, CCR8, GITR, and BATF (Figure 6J). These results position STAT4 as a central transcriptional regulator of GITR⁺ Treg identity and immunosuppressive programming.

### 2.9 Therapeutic Potential of Combining MET Inhibition with GITR Agonism

To evaluate the therapeutic relevance of targeting the MET–GITR⁺ Treg axis, we employed an orthotopic KPC‐derived PDAC mouse model. Based on prior evidence that GITR agonism can impair Treg‐mediated immunosuppression while enhancing effector T cell activity, we administered the GITR agonist DTA‐1 in combination with the MET inhibitor INC280.^[^ ^25^, ^27^ ^]^

**Figure 7:**
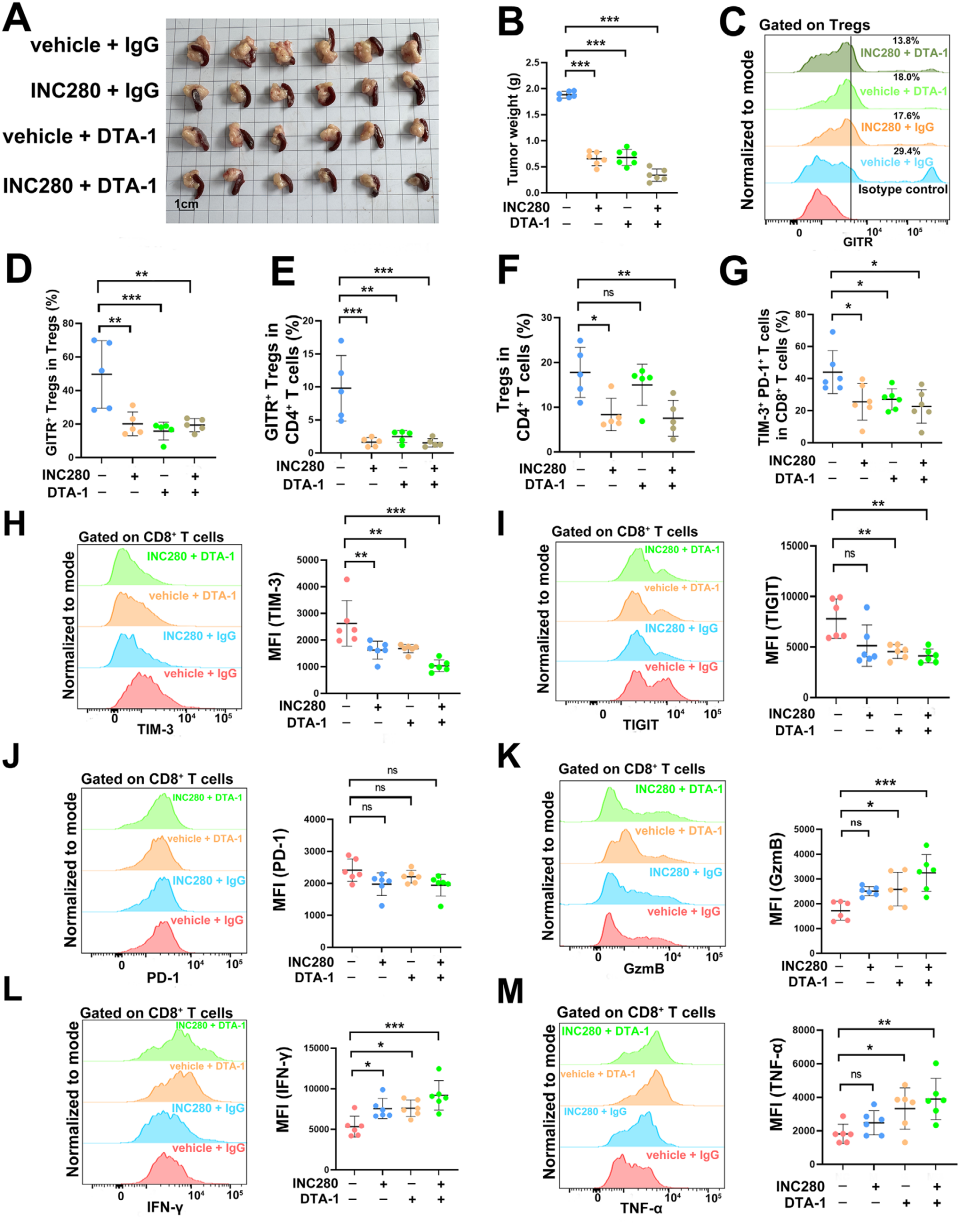
MET inhibitor and GITR agonism combination suppresses PDAC progression A) Orthotopic inoculated KPC tumors from the control, INC280 monotherapy, DTA‐1 monotherapy and combined therapy groups. B) Quantification of tumor weights under different treatment. *n* = 6 per group. ^***^ *p* < 0.001. C,D) Representative histogram images and quantification of GITR^+^ Tregs frequency among Tregs with indicated treatments. *n* = 5 per group. ^**^ *p* < 0.01, ^***^ *p* < 0.001. (E) Quantification of GITR^+^ Tregs frequency among CD4^+^ T cells with indicated treatments. *n* = 5 per group. ^**^ *p* < 0.01, ^***^ *p* < 0.001. F) Quantification of Tregs frequency among CD4^+^ T cells under different treatment. n = 5 per group. ns, not significant, ^*^ *p* < 0.05, ^**^ *p* < 0.01. G) Flow cytometry analysis of TIM‐3^+^PD‐1^+^ T cells frequency among CD8^+^ T cells with indicated treatments. *n* = 6 per group. ^*^ *p* < 0.05. H–J) Flowcytometry analysis of TIM‐3 (H), TIGIT (I) and PD‐1 (J) expression in CD8^+^ T cells with indicated treatments. n = 6 per group. ns, not significant, ^*^ *p* < 0.05, ^**^ *p* < 0.01, ^***^ *p* < 0.001. K–M) Flowcytometry analysis of GzmB (K), IFN‐γ (L) and TNF‐α (M) expression in CD8^+^ T cells under different treatment. *n* = 6 per group. ns, not significant, ^*^ *p* < 0.05, ^**^ *p* < 0.01, ^***^ *p* < 0.001.

Mice were randomized into four treatment groups: 1) control (vehicle + IgG), 2) INC280 monotherapy (MET inhibitor + IgG), 3) DTA‐1 monotherapy (vehicle + GITR agonist), and 4) combination therapy (INC280 + DTA‐1). Both monotherapies significantly reduced tumor burden relative to controls, while the combination therapy yielded the most substantial tumor suppression (**Figure** **7**A,B). To assess immunologic mechanisms, we performed flow cytometry on immune cells. Both INC280 and DTA‐1 significantly reduced GITR⁺ Treg frequency among total Tregs and CD4⁺ T cells (Figure 7C–E). Interestingly, only INC280 reduced the total Treg population within the CD4⁺ compartment, while combination therapy did not further enhance Treg depletion (Figure 7F), suggesting that MET inhibition and GITR agonism operate via distinct and non‐redundant mechanisms.

Phenotypic analysis of CD8⁺ T cells revealed differential impacts on exhaustion and effector function. All treatment arms significantly reduced TIM‐3⁺PD‐1⁺ exhausted CD8⁺ T cells (Figure 7G). DTA‐1 monotherapy decreased TIGIT and TIM‐3 expression, while INC280 selectively downregulated TIM‐3, without significant effects on TIGIT or PD‐1 (Figure 7H–J). Importantly, combination therapy led to marked increases in cytotoxic markers of CD8⁺ T cells, including GzmB, IFN‐γ, and TNF‐α (Figure 7K–M), alongside a reduction in exhaustion markers, indicating enhanced functional reprogramming of cytotoxic T cells.

Taken together, these findings demonstrate that MET inhibition and GITR agonism synergize to enhance antitumor immunity in PDAC. While INC280 primarily acts by targeting the immunosuppressive Treg compartment, DTA‐1 functions to restore CD8⁺ T cell effector capacity and alleviate exhaustion. The combination therapy exerts additive immunomodulatory effects, resulting in superior tumor control driven by cooperative reactivation of effector T cell responses.

## 3 Discussion

PD‐1 plays an irreplaceable roles in driving T cell exhaustion. Blocking PD‐1 from binding to its ligand has been shown to prolong survival in patients with various cancers. However, tumor‐intrinsic PD‐1 was also found in tumor cells and exhibited its pro‐tumoral effect in various cancers, including PDAC.^[^ ^9^, ^10^, ^34^ ^]^ As a previous study has detailed reported that tumor‐intrinsic PD‐1 could activate the MET signaling in PDAC cells to promote growth, migration, and invasion.^[^ ^11^ ^]^ In this study, we elucidated that tumor‐intrinsic PD‐1 is clinically correlated with MET activation, thereby fostering an immunosuppressive TME, which occurs, in part, through the accumulation of GITR^+^ Tregs in PDAC. Our founding highlight that the tumor‐promoting effects of intrinsic PD‐1 extend beyond directly influencing cancer cells to shape a more tolerogenic environment through the PD‐1/MET axis.

MET, a receptor tyrosine kinase (RTK), is activated by ligands such as hepatocyte growth factor (HGF)/scattering factor (SF) and drives key processes in tumor progression, including invasion, proliferation, and angiogenesis. While its impact on cancer cells is well characterized, its effects on the TIME are less understood. In a previous study, MET can shift macrophages from an M1 to M2 phenotype.^[^ ^35^ ^]^ However, we observed no significant differences in macrophage between MET^high^ or MET^low^ groups in PDAC. This indicates the unique role of MET signaling in this particular cancer type. In this study, a positive correlation between Tregs infiltration and MET activation was observed. Distinctive expression of immune checkpoint molecules, including PD‐1, TIGIT, GITR and CTLA‐4, has been found in Treg cells, which manifests different phenotype and subtype of Treg cells in TME.^[^ ^36^ ^]^ In our study, MET activation specifically promotes GITR^+^ Tregs accumulation, a subset known for heightened suppressive activity.^[^ ^25^ ^]^ High levels of GITR^+^ Tregs are linked to poor prognosis in pancreatic cancer, suggesting that MET signaling could promote immunosuppression within the TME by reprogramming intratumoral Tregs evolution. These findings underscore the potential therapeutic value of targeting MET in PDAC.

Treg cells are crucial immunosuppressive cells that dampen both innate and adaptive immune responses. In this study, the presence of lymph node metastasis (N stage) was the only clinicopathological factor significantly associated with the accumulation of GITR^+^ Tregs. This finding aligns with growing evidence that an immunosuppressive TME can support metastasis by enhancing tumor cell movement through the release of specific cytokines.^[^ ^37^, ^38^, ^39^, ^40^ ^]^ Furthermore, capable of spreading more readily to lymph nodes can, in turn, recruit additional Tregs into the TME.^[^ ^41^ ^]^ Taken together, these observations raise the question of whether the accumulation of GITR^+^ Tregs directly drives tumor metastasis or disease progression, warranting further investigation.

Interestingly, our study revealed that patients with elevated infiltration of GITR⁻ Tregs tended to have longer OS and RFS. This pattern is consistent with findings in lung, colorectal, and gastric cancers, where increased Treg presence also correlated with improved prognosis.^[^ ^42^, ^43^, ^44^, ^45^ ^]^ One plausible reason is that Tregs can moderate the inflammation triggered by certain microbes—an effect particularly relevant in the gastrointestinal tract.^[^ ^45^, ^46^ ^]^ However, the pancreas hosts far fewer microbes than the stomach or colon, which may influence the immunological dynamics in this setting.^[^ ^47^ ^]^ It is worth noting that Alexander et al. categorized the TME into four subtypes–IE/F (Immune‐Enriched/Fibrotic), IE (Immune‐Enriched/Nonfibrotic), F (Fibrotic), and D (Immune‐Depleted)– and noted that the IE subtype is generally associated with better outcomes. Still, IE‐type TMEs contain both anti‐tumor and pro‐tumor immune components. In contrast, D‐type TMEs lack both, indicating that anti‐tumor responses frequently coexist with immunosuppressive elements. Other classification systems also show that active anti‐tumor immunity often appears alongside immunosuppressive features.^[^ ^48^, ^49^, ^50^ ^]^ Specifically, Peter et al.'s classification of the PDAC TME, for example, identified an “Immunogenic” subtype characterized by the enrichment of both Tregs and CD8⁺ T cells, suggesting a complex interplay of immune activation and suppression.^[^ ^51^ ^]^ Together, these findings imply that the presence of GITR⁻ Tregs may reflect an actively engaged intratumoral immune response rather than an environment devoid of immune activity.^[^ ^46^ ^]^

Our findings indicate that GITR^+^ Treg infiltration provides a meaningful way to stratify patient survival. Notably, among patients with high GITR^+^ Treg levels, the commonly used PDAC biomarker CA19‐9 loses its prognostic relevance. While CA19‐9 generally reflects the tumor's intrinsic malignant potential, GITR^+^ Treg infiltration highlights the immunosuppressive character of the TME.^[^ ^52^ ^]^ In highly immunosuppressive environments, these external factors appear to exert a stronger influence on patient outcomes than the tumor's inherent biology. By leveraging tumor‐infiltrating GITR^+^ Treg as a novel biomarker, clinicians can more accurately assess survival prospects for patients whose prognoses are not adequately captured by serum CA19‐9. Incorporating this measure could help refine personalized treatment approaches, taking into account both the tumor's underlying characteristics and the impact of the immunosuppressive microenvironment.

Further analysis of scRNA‐seq data revealed that GITR^+^ Tregs function as effector cells, whereas KLF2^+^ Tregs remain in a resting state. Notably, GITR⁺ Tregs exhibit significantly stronger immunoregulatory capabilities than KLF2⁺ Tregs. Although GITR is traditionally seen as a constitutive regulatory molecule on all Tregs and a potential therapeutic target, our scRNA‐seq analysis and PDAC tissue staining demonstrated that GITR expression is restricted to a specific Treg subset, not universally expressed.^[^ ^53^ ^]^ This suggests that GITR can serve as a marker to distinguish highly immunosuppressive Tregs from those with weaker regulatory functions. Additionally, within the GITR⁺ Treg population, GITR expression varies. Pseudo‐time trajectory analysis revealed that higher GITR levels correlate with a more terminally differentiated state of Tregs, characterized by elevated expression of FoxP3 and BATF. Therefore, GITR not only identifies Tregs with strong immunoregulatory capacity but also provides insight into their maturation from naïve to fully differentiated states.^[^ ^25^ ^]^ These findings open the door to therapeutic strategies that selectively target GITR^+^ Tregs, particularly those with high GITR expression (GITR^hi^ Tregs), to curb immunosuppression while preserving less suppressive Treg subsets. The selective depletion of GITR⁺ Tregs, as observed in our preclinical models, offers a therapeutic advantage by potentially mitigating the systemic immune‐related side effects typically associated with broad Treg depletion. These findings support a rational strategy to co‐target MET and GITR⁺ Tregs as a means to reshape the immunosuppressive TME in PDAC. However, further investigation is necessary to evaluate the safety, durability, and clinical feasibility of this combinatorial approach before translation into therapeutic protocols.

Our study also advances understanding of the mechanisms driving GITR⁺ Treg accumulation within the TME. While emerging evidence supports an immunomodulatory role for IL‐23 in stabilizing tumor‐infiltrating Tregs,^[^ ^54^, ^55^, ^56^ ^]^ the role of IL‐23 in PDAC remains largely undefined. Building upon this foundation, we present the first evidence that IL‐23–driven STAT4 phosphorylation reprograms Tregs by promoting their phenotypic conversion into effector GITR⁺ Treg subsets with enhanced immunosuppressive function. Intriguingly, IL‐23R expression has been documented across multiple immune lineages, including T cells, macrophages, and neutrophils—key players in sculpting the PDAC immune landscape.^[^ ^54^, ^57^, ^58^ ^]^ This pleiotropic expression pattern suggests that IL‐23 may act as a central coordinator of multicellular crosstalk within the TME, underscoring the need for systematic mapping of IL‐23–responsive signaling networks in PDAC.

Our bioinformatic and in vitro analyses converged on STAT4 as a pivotal transcription factor driving GITR⁺ Treg development. Notably, STAT4 exhibits a distinct expression and activation pattern compared to canonical Treg‐associated factors, such as FoxP3 and BATF. STAT4 is canonically activated by cytokines IL‐12 and IL‐23,^[^ ^31^, ^59^ ^]^ and while early studies suggested that STAT4 phosphorylation undermines Treg suppressive function by inducing a Th1‐like phenotype that promotes anti‐tumor immunity,^[^ ^60^, ^61^ ^]^ emerging data reveal a more nuanced regulatory role. Recent studies have delineated a biphasic paradigm of p‐STAT4 activity in Treg plasticity. For instance, Diana et al.^[^ ^33^ ^]^ demonstrated that low‐dose IL‐12–mediated STAT4 activation in peripheral resting Tregs promotes their differentiation into T follicular regulatory (Tfr) cells, marked by increased expression of key effector molecules such as CTLA‐4, CD25, and GITR. Conversely, high‐dose IL‐12 stimulation results in the loss of regulatory phenotype, leading to diminished suppressive function.^[^ ^62^, ^63^ ^]^ These findings suggest that STAT4 acts as a rheostat‐like modulator of Treg functional states, with outcomes determined by cytokine concentration gradients within the tissue microenvironment.

Despite these insights, our study has several limitations. First, the prognostic significance of GITR⁺ Tregs was assessed in a single‐center retrospective cohort, which may limit generalizability. Future investigations incorporating prospective, multi‐center validation are warranted to strengthen the robustness of these observations. Second, although our findings establish a functional link between STAT4 activation and GITR⁺ Treg accumulation, the precise molecular mechanisms underlying STAT4‐mediated immunosuppression require further elucidation, including potential chromatin‐level regulation and cytokine‐dependent signaling dynamics. These questions represent important avenues for future research.

## 4 Conclusion 

This study identifies the tumor‐intrinsic PD‐1/MET signaling axis as a key driver of immunosuppressive remodeling in the PDAC microenvironment through the IL‐23/STAT4‐dependent accumulation of GITR⁺ Tregs. Compared to conventional Tregs, GITR⁺ Tregs exhibit stronger prognostic relevance and enhanced immunosuppressive capacity, underscoring their unique functional role within the tumor milieu. Collectively, our findings highlight MET signaling and GITR⁺ Tregs as actionable targets to counteract immune evasion and improve the efficacy of immunotherapeutic strategies in PDAC.

## 5 Experimental Section

### Study Cohort and Tissue Microarray (TMA)

This study included two cohorts of patients diagnosed with PDAC. The first cohort comprised 29 patients who underwent surgical resection and received a pathological diagnosis of PDAC at Zhongshan Hospital, Fudan University, between September 2018 and October 2019. The second cohort included an additional 116 patients diagnosed with PDAC from November 2020 to December 2021. The inclusion and exclusion criteria were: 1) histological diagnosis of PDAC with complete R0 resection; 2) availability of comprehensive clinical and follow‐up data; 3) no history of other primary malignancies; 4) no preoperative neoadjuvant therapies or participation in any clinical trial involving immune or targeted therapies; 5) no evidence of distant metastasis. These criteria ensured the selection of a homogeneous patient population, facilitating accurate analysis of the prognostic factors under investigation. The study protocol was approved by the Institutional Review Board and Ethics Committee of Zhongshan Hospital, Fudan University (No. B2024‐052).

Tumor tissues were fixed in formalin and embedded in paraffin. An experienced pathologist selected representative tumor regions, which were then sectioned into 2.0 mm diameter cylindrical samples for the production of TMA. Comprehensive clinicopathological data—including age, sex, tumor site, preoperative serum carbohydrate antigen 19‐9 (CA19‐9) levels, American Joint Committee on Cancer (AJCC) eighth edition of TNM stage, tumor differentiation, presence of microvascular and perineural invasion, postoperative adjuvant radiotherapy (ART) and chemotherapy (ACT), and follow‐up information—were collected by one surgeon and verified by another.

### Survival Follow‐Up

The final follow‐up for this study was completed in May 2024. After undergoing surgical resection, all participants were regularly monitored in accordance with previously established protocols. Overall survival (OS) was determined as the duration from the date of surgery to death from any cause, censoring, or the last recorded follow‐up in May 2024. Recurrence‐free survival (RFS) was defined as the period from surgery to the first instance of tumor relapse or death, or the last follow‐up, whichever occurred first. These definitions are consistent with those employed in the earlier publications.^[^ ^20^ ^]^

### Cell Culture

Human PDAC cell lines (PANC‐1, ASPC‐1, BxPC‐3, CFPAC‐1, and MIA‐PaCa‐2) were obtained from the National Collection of Authenticated Cell Cultures, and murine PDAC cell line KPC (LSL‐Kras^G12D/+^; Trp53^R172H/+^; Pdx‐1‐Cre) were provided by Johns Hopkins Hospital. PANC‐1, MIA‐PaCa‐2, and KPC cells were cultured in high glucose DMEM supplemented with 10% fetal bovine serum (FBS). CFPAC‐1 cells were maintained in IMDM with 10% FBS, while BxPC‐3 and ASPC‐1 cells were cultured in RPMI‐1640 with 10% FBS. Human Tregs were cultured in X‐VIVO medium supplemented with 10% FBS, 1% GlutaMAX, 1% sodium pyruvate, and 1% MEM non‐essential amino acids, along with IL‐2 (20ng mL^−1^; UA BIOSCIENCE, UA040171), and TGF‐β (5ng mL^−1^; UA BIOSCIENCE, UA040085). iTregs were incubated with conditioned medium (CM) from human PDAC cell lines for 48 h before analysis. All cell lines were maintained at 37 °C in a humidified incubator with 5% CO_2_, and were routinely tested for mycoplasma contamination every three days.

### Lentivirus Transfection

To generate MET‐overexpressing (MET^OE^) cell lines, recombinant lentiviral constructs were acquired from GeneCard (China). The coding sequence of MET was cloned into the GV341 lentiviral vector (Ubc‐MCS‐3FLAG‐SV40‐puromycin) for stable expression. Lentiviral particles were packaged according to the manufacturer's instructions. PDAC cells were transduced with the MET‐expressing lentivirus, followed by puromycin selection (concentration per manufacturer's guidelines) to eliminate non‐transduced cells, as previously published.^[^ ^10^ ^]^ Successful overexpression of MET was confirmed by western blot analysis.

### Immunofluorescence (IF) and Immunohistochemical (IHC) Staining

Formalin‐fixed, paraffin‐embedded (FFPE) tissue specimens from the TMAs were utilized for IF staining. To identify GITR^+^CD4^+^FoxP3^+^ Tregs and IL‐23R^+^ Tregs, the following monoclonal antibodies: anti‐CD4 (Maxim, RMA‐0620), anti‐FoxP3 (Cell Signaling Technology, 98377), anti‐GITR (Abcam, ab223841), was employed and IL‐23R (Abmart, PS06333S). The antibodies were incubated with hydrated TMA sections overnight at 4°C, followed by labeling with Alexa Fluor 647, Alexa Fluor 555, and Alexa Fluor 488 fluorophores. For evaluating tumoral IL‐23 expression, anti‐panCK (CST, 4545) and anti‐IL‐23 (Abcam, ab7753) antibodies were used for staining. Anti‐CD8 (Abcam, 237709) and anti‐Ki67 (Abcam, ab1667) antibodies were used to determine proliferating CD8^+^ T cells. Anti‐TIM‐3 (Abcam, ab241332) and anti‐PD‐1 (CST, 86163) antibodies were used for staining TIM‐3^+^PD‐1^+^CD8^+^ T cells. Anti‐GzmB (Abcam, ab208586) and anti‐Perforin (Absin, 135524) antibodies were used for CD8^+^ T cell cytotoxicity assessment. Subsequently, the TMAs were counterstained with DAPI for 5 min to visualize cell nuclei. After thorough washing and mounting, the slides were scanned using a 3D HISTECH Pannoramic SCAN fluorescence scanner. For each TMA spot, three independent and representative high‐power fields (HPF) were captured at ×200 magnification (0.305 mm^2^ per field). Two independent pathologists reviewed each selected field to accurately quantify the number of total Tregs and GITR^+^ Tregs.

For IHC staining, monoclonal antibodies against PD‐1 (Abcam, ab52587) was utilized, MET (STARTER, S0B2052), and p‐MET (Abcam, ab68141) to evaluate the expression levels of tumor‐intrinsic PD‐1 and p‐MET in each patient. The expression of MET, p‐MET, and PD‐1 in TMA specimens was independently evaluated by two board‐certified pathologists blinded to the treatment information. Immunoreactivity scores were calculated by multiplying the percentage of positively stained tumor cells by the staining intensity, yielding a final score ranging from 0 to 12. Based on prior publications,^[^ ^10^, ^64^ ^]^ samples with a score ≥6 were categorized as high expression, while those with a score <6 were classified as low expression. For quantitative analysis in dot plots, three representative fields per specimen were selected. Integrated Optical Density (IOD) was measured using ImageJ software, applying standardized thresholding to distinguish specific signals from the background. The average IOD from the three fields was used to generate a composite expression score for each marker. The IHC staining protocol for immune cell markers has been described in detail in this previous studies.^[^ ^22^, ^65^ ^]^

### Flow Cytometry

In our previous report, fresh surgical PDAC tissues and utilized flow cytometry was dissociated to characterize the phenotypes of tumor‐infiltrating CD8⁺ T cells. Specifically, for cytotoxic markers (TNF‐α, IFN‐γ, Granzyme B, and Perforin) was stained and exhaustion markers (PD‐1, CTLA‐4, TIM‐3, and TIGIT).^[^ ^20^ ^]^ Building on these findings, IHC staining was further employed on FFPE slides from the same cases to evaluate the expression levels of p‐MET. This comprehensive approach allowed us to explore the interplay between MET activation and the functional status of tumor‐infiltrating CD8⁺ T cells, providing deeper insights into the immunosuppressive mechanisms within the PDAC microenvironment.

For in vitro Tregs flow cytometry, human Tregs were stimulated with CM from PDAC cell lines for 48 h, then harvested and washed with PBS. Cells were first stained surface markers for 30 min at 4 °C, followed by fixation using IC Fixation Buffer (Invitrogen, FB001). After three PBS washes, cells were permeabilized with Permeabilization Buffer (Invitrogen, 88‐8824‐00) for 30 min, then stained for intracellular markers. The following fluorochrome‐conjugated antibodies were used for in vitro Treg analysis: Anti‐CD3e (BD Pharmingen, BV510), anti‐CD4 (BD Pharmingen, FITC), anti‐FoxP3 (BD Pharmingen, Alex Flour 647), anti‐GITR (BD Pharmingen, BV421), anti‐pSTAT4(BD Phosflow, PE‐Cy7), anti‐CD39 (BD Pharmingen, FITC) and anti‐CTLA‐4 (BD Pharmingen, PE).

For in vivo immune cell analysis, tumor tissues were dissociated into single‐cell suspensions using the Tumor Dissociation Kit (Absin, abs9482) per the manufacturer's protocol. Viable cells were labeled using BD Horizon™ Fixable Viability Stain 780 (BD Pharmingen, APC‐Cy7) for 15 min at room temperature. Both in vitro and in vivo samples were processed using the same surface and intracellular staining workflow. For in vivo Treg profiling, the following antibodies were used: Anti‐CD45 (BD Pharmingen, PerCP‐Cy5.5), anti‐CD3e (BD Pharmingen, FITC), anti‐CD4 (BD Pharmingen, BB700), anti‐FoxP3 (BD Pharmingen, PE), and anti‐GITR (BD Pharmingen, PE‐Cy7). For CD8⁺ T cell exhaustion analysis, the following antibodies were used: Anti‐CD45 (BioLegend, FITC), anti‐CD8a (BioLegend, APC), anti‐TIGIT (BioLegend, PE), anti‐PD‐1 (BioLegend, PerCP‐Cy5.5) and anti‐TIM‐3 (BioLegend, PE‐Cy7). For CD8⁺ T cell cytotoxicity analysis, the following antibodies were used: Anti‐GzmB (BioLegend, PerCP‐Cy5.5), anti‐TNF‐α (BioLegend, BV510), and anti‐IFN‐γ (BioLegend, PE‐Cy7). All flow cytometry data were acquired using a BD FACSAria™ Flow Cytometer (BD Biosciences, USA) and analyzed with FlowJo™ v10 software (BD Biosciences, USA).

### In Vivo Animal Experiment

Male C57BL/6 mice (6–7 weeks old) were maintained under specific pathogen‐free (SPF) conditions. Orthotopic pancreatic tumors were established by intrapancreatic injection of 5 × 10⁴ KPC cells suspended in a 1:1 (v/v) mixture of Matrigel. Once tumors reached an average volume of≈300 mm^3^, mice were randomized into four treatment groups: 1) vehicle (5% DMSO + 45% PEG300/5% Tween80 + 50% ddH_2_O) + IgG control, 2) MET inhibitor monotherapy: INC280 (10 mg kg day^−1^ for 8 days, MedChemExpress) + IgG, 3) GITR agonist monotherapy: DTA‐1 (100 µg mouse day^−1^ for 4 days, BioXCell) + vehicle, and 4) combination therapy: INC280+ DTA‐1. INC280 was formulated in a vehicle solution of DMSO/PEG300/Tween80/ddH₂O (5:45:5:50, v/v) and administered by intraperitoneal injection. DTA‐1 was reconstituted in ultrapure water and also administered intraperitoneally. Following 20 days of treatment, mice were euthanized, and tumors were harvested for analysis of GITR^+^ Tregs (*n* = 5 per group), and tumor weight measurement and tumor‐infiltrating CD8^+^ T cell assessment (*n* = 6 per group). All procedures were conducted in accordance with protocols approved by the Animal Ethics Committee of Zhongshan Hospital, Fudan University.

### RNA Sequencing

CFPAC‐1 cells were treated with or without INC280 (20nm) for 4 days prior to RNA extraction. Total RNA was isolated using the Total RNA Isolation Kit (Epizyme, YY101) following the manufacturer's instructions. RNA quality control, library preparation, and sequencing were performed by Oebiotech Co., Ltd. (Shanghai, China). Raw sequencing reads were demultiplexed and converted to FASTQ format using bcl2fastq v2.20 (Illumina). Reads were aligned to the GRCh38 reference genome, and transcript abundance was quantified. Differential gene expression analysis was performed using the DESeq2 package, following standard workflows.

### Western Blot Analysis

Total protein was extracted from tumor cells using RIPA lysis buffer supplemented with protease and phosphatase inhibitors. Protein concentrations were determined using a BCA Protein Assay Kit. Equal amounts of protein were separated by SDS‐PAGE and transferred onto polyvinylidene difluoride (PVDF) membranes. embranes were blocked in 5% non‐fat milk in TBS‐T (Tris‐buffered saline with 0.1% Tween‐20) for 1 hour at room temperature, followed by overnight incubation at 4 °C with primary antibodies. After washing, membranes were incubated with appropriate HRP‐conjugated secondary antibodies for 1 hour at room temperature. Protein bands were visualized using an enhanced chemiluminescence (ECL) detection system.^[^ ^10^ ^]^

### Quantitative Real‐Time PCR (qRT‐PCR)

CFPAC‐1 was treated with or without INC280 (20nm) for 4 days prior to RNA extraction. Total RNA was isolated and reverse transcribed into cDNA using the RevertAid First Strand cDNA Synthesis Kit (Thermo Fisher Scientific, K1691), according to the manufacturer's instructions. Quantitative PCR was performed using SYBR Green Master Mix and gene‐specific primers on the QuantStudio™ 5 Real‐Time PCR System (Applied Biosystems, USA). GAPDH was used as the internal control, and relative gene expression levels were calculated using the 2^–ΔΔCt method.^[^ ^10^ ^]^ Primer sequences are provided in Table S4 (Supporting Information).

### Enzyme‐Linked Immunosorbent Assay (ELISA)

The secretion of IL‐10 and TGF‐β by Tregs was measured using Human IL‐10 ELISA and Human TGF‐β ELISA kits (Feiyuebio, China), following the manufacturer's protocols. Tregs were first incubated with CM from CFPAC‐1 cells, treated with or without INC280 (20 nm), for 48 h. After washing with PBS to remove residual CM, cells were resuspended in fresh culture medium at a density of 2 × 10⁶ cells mL^−1^ and cultured for an additional 72 h. Supernatants were then collected for ELISA analysis. For IL‐23 detection, CFPAC‐1 cells were treated with 20 nm INC280 or vehicle control for 96 h. Culture supernatants were harvested and analyzed using the Human IL‐23 ELISA kit (Feiyuebio, China), according to the manufacturer's instructions.

Optical density (OD) was measured at 450 nm using a microplate reader (Molecular Devices, China). Cytokine concentrations were calculated based on a standard curve generated for each analyte.

### In Vitro Expansion of Human Tregs

Naïve CD4^+^ T lymphocytes (CD4^+^ CD25^low^ CD127^high^ CD45RA^+^ phenotype) were isolated from peripheral blood mononuclear cells (PBMCs) of healthy donors using a BD FACSAria III cell sorter (BD Biosciences, USA). Sorted cells were cultured under inducible Treg (iTreg)–polarizing conditions in X‐VIVO medium (Lonza, 04‐418Q) supplemented with 10% FBS (Servicebio, G8003), 1% sodium pyruvate, 1% MEM non‐essential amino acids, and 1% GlutaMAX in the presence of recombinant human IL‐2 (20ng mL^−1^; UA BIOSCIENCE, UA040171), and TGF‐β (5ng mL^−1^; UA BIOSCIENCE, UA040085). For T cell activation, anti‐human CD3 monoclonal antibody (STARTER, S0B0009) and anti‐human CD28 monoclonal antibody (STARTER, S0B0010) were added to the culture. After 7 days of induction, the frequency of FoxP3⁺ cells exceeded 90%, confirming successful Treg differentiation. These cells were then used for downstream analyses.

### RNA Expression and Clinical Data Collection

Bulk RNA sequencing data for 178 pancreatic cancer patients were obtained from the TCGA cohort (https://cancergenome.nih.gov/), RNA microarray data for 233 patients from the ICGC‐PACA cohort (https://dcc.icgc.org/), and RNA microarray data for 288 pancreatic cancer patients from the E‐MTAB‐6134 cohort (https://www.ebi.ac.uk/biostudies/arrayexpress). Corresponding clinical prognostic data, including survival status, OS, recurrence status, and RFS, were also collected for these cohorts. Additionally, RNA sequencing data for normal pancreatic tissues were retrieved from the GTEx database (https://www.genome.gov/Funded‐Programs‐Projects/Genotype‐Tissue‐Expression‐Project).

### Single‐Cell RNA Sequencing Data Clustering and Annotation

Processed single‐cell RNA sequencing (scRNA‐seq) data was downloaded from the GSE155698 dataset available on the GEO database (https://www.ncbi.nlm.nih.gov/geo/query/acc.cgi?acc=GSE155698).^[^ ^66^ ^]^ Downstream analysis of this scRNA‐seq data was performed using Seurat (v5.1.0) in R (v4.2.2).^[^ ^67^ ^]^ Sequencing data from 16 pancreatic cancer patients in the GSE155698 dataset were integrated, filtering out cells with mitochondrial gene content greater than 20% and with ‘nFeature_RNA’ less than 200, genes expressed in less than 3 cells. After data normalization, principal component analysis (PCA) was performed using the top 2500 highly variable genes, followed by batch effect correction across samples using “RunHarmony”. Based on Harmony‐reduced dimensions, uniform manifold approximation and projection (UMAP) was applied for dimensionality reduction. Cell clustering was conducted using “FindNeighbors” and “FindClusters” function, based on Harmony reduction. Differentially expressed genes (DEGs) were identified using “FindAllMarkers” function, and cell types were annotated according to well‐established biomarkers. However, certain cell populations, such as CD4^+^ T cells and Tregs within the T cell cluster, as well as macrophages and dendritic cells within the myeloid cell cluster, were not distinctly separable. To address this, additional sub‐clustering and annotation within the T cell and myeloid cell populations were performed. This refined approach allowed us to accurately differentiate specific cell types, including Tregs, dendritic cells, and monocytes.

### inferCNV Analysis

To differentiate malignant epithelial cells from their normal counterparts within our scRNA‐seq data, the inferCNV tool (https://github.com/broadinstitute/inferCNV) utilized. This tool is widely recognized for identifying abnormal chromosomal copy number variations (CNVs) that are indicative of tumor cells. In this study, the R package inferCNV (v1.21.0) was utilized to analyze all ductal cells. Mast cells were chosen as the reference normal cell type to serve as a baseline for the inferCNV analysis.

### AddModuleScore

AddModuleScore was employed to calculate module scores for feature gene expression programs in single cells. Specifically, the activation of the MET pathway was calculated for each ductal cell in the GSE155698 dataset using AddModuleScore function in Seurat, and a t‐test was conducted to compare module scores of MET pathway between MET^high^ and MET^low^ group. Inhibitory cytokines (TGF‐β, IL‐10, IL‐35, FGL2), metabolism disruption (CD25, CD39, CD73), cytolysis (PRF1, GZMB, FASLG), and targeting on APC (LAG‐3, CTLA‐4, Galectin‐1, Galectin‐10, NRP1) module scores of Tregs were also calculated using AddModuleScore algorithm. These four biological pathways of Tregs and corresponding signatures were concluded according to previously published studies.^[^ ^29^ ^]^

### Psuedo‐Time Analysis

Pseudo‐time analysis was conducted using the monocle (v = 2.26) package in R. The diff_test_res function was applied to identify DGEs between GITR^+^ Tregs and KLF2^+^ Tregs. Genes with q‐value < 0.05 were selected to construct the pseudo‐time trajectory. Additionally, the top 1,000 highly variable genes within the Treg population were utilized with “plot_pseudotime_heatmap” function in monocle to generate a heatmap, with num_clusters set to 3. Enrichment analysis was conducted using “enricher” function in R package ClusterProlifer. Transcription factor prediction was conducted by ChEA3, incorporating the DEGs between KLF2^+^ Tregs and GITR^+^ Tregs.^[^ ^68^ ^]^

### Gene Set Variation Analysis (GSVA)

GSVA was performed using the GSVA R package (v1.46.0) to assess the activation status of the MET signaling pathway across samples from the TCGA, ICGC, and E‐MTAB‐6134 cohorts. The analysis was based on the BIOCARTA_MET_PATHWAY gene set retrieved from the MSigDB database (https://www.gsea‐msigdb.org/gsea/msigdb/human/geneset/BIOCARTA_MET_PATHWAY.html).

For the GSE155698 scRNA‐seq dataset, expression matrices of malignant epithelial cells were aggregated at the patient level using the AverageExpression function (Seurat package) to generate pseudo‐bulk profiles, reducing technical noise while preserving biological variability. GSVA was then applied to calculate MET activation scores for each patient. Patients were stratified into MET^low^ (GSVA score < 0; T1, T3, T5, T9, T13, T15, T16) and MET^high^ (GSVA score > 0; T2, T4, T6, T7, T8, T10, T11, T12, T14) groups

In the Treg subset of the GSE155698 scRNA‐seq dataset, the top 10 upregulated genes (ranked by p‐value, log₂FC > 1) in GITR⁺ Tregs versus KLF2⁺ Tregs were identified and defined as the GITR⁺ Treg gene signature. This signature was then applied to the TCGA, ICGC, and E‐MTAB‐6134 bulk transcriptomic datasets using GSVA to infer GITR⁺ Treg infiltration scores across patient samples.

### Gene Set Enrichment Analysis (GSEA)

GSEA was conducted using package clusterProfiler (v = 4.6.2) in R. Gene sets were acquired from MSigDB database (https://www.gsea‐msigdb.org/gsea/msigdb/download_file.jsp?filePath=/msigdb/release/2024.1.Hs/c2.cp.v2024.1.Hs.entrez.gmt; https://www.gsea‐msigdb.org/gsea/msigdb/download_file.jsp?filePath=/msigdb/release/2024.1.Hs/c5.go.v2024.1.Hs.entrez.gmt).

### xCell and ABSOLUTE Analysis

xCell algorithm is based on single‐sample GSEA (ssGSEA) and is used to infer the abundance of immune and stromal components from bulk RNA‐seq or microarray data. Specifically, xCell (R package xCell, v1.1.0; https://comphealth.ucsf.edu/app/xcell) was used to estimate Treg infiltration in the TCGA and E‐MTAB‐6134 cohorts. In parallel, tumor purity was assessed using ABSOLUTE, implemented via the DoAbsolute function in the ABSOLUTR R package. The cutoff for ABSOLUTE‐defined high tumor purity was based on thresholds previously validated by Andrew et al.^[^ ^17^ ^]^

### Statistical Analysis

Statistical analyses were performed using SPSS version 21.0 and R version 4.2.2. The optimal cutoff values for continuous variables, including MET pathway scores and GITR^+^ Tregs infiltration scores, were determined by “surv_cutpoint” function from the “survminer” R package (v0.4.9), with the parameter “minprop” set to 0.25 to ensure a minimum proportion in each group. Associations between p‐MET expression or GITR^+^ Treg infiltration and clinicopathological features were assessed using Pearson's Chi‐square test or Fisher's exact test, as appropriate. For comparisons involving continuous variables grouped by categorical factors, Student's *t*‐test or the the Mann‐Whitney *U* test was applied based on data distribution. Survival analysis was performed using Kaplan–Meier curves, which were compared with log‐rank tests. Independent prognostic factors for OS and RFS were identified using univariate and multivariate Cox proportional hazards models. For comparisons of quantitative data between two groups, a two‐tailed Student's t‐test was applied. For multiple group comparisons, one‐way ANOVA was used, followed by LSD or Tamhane's post hoc tests, depending on variance homogeneity. A p‐value < 0.05 was considered statistically significant.

## Conflict of Interest

The authors declare no conflict of interest.

## Author Contributions

J.H., H.Y., T.H., J.H., and N.P. contributed equally as co‐first authors of this article. J.H., H.Y., T.H., J.H., and Z.J. data acquisition, data analysis, statistical analysis, manuscript writing. Q.C., Z.X., Y.X., Y.X., H.W., W.W., W.W., Y.J., and W.L. data acquisition, manuscript review. N.P., J.Y., W.L., and L.L. acquired funding acquisition, data acquisition, data analysis, manuscript review. N.P. supervised the study. All authors read and approved the final version of the manuscript. L.L. is responsible for the overall content as guarantor.

## Ethics Approval

The protocol of this study has been approved by the institutional review board and ethics committee of Zhongshan Hospital, Fudan University (B2024‐052). Written informed consent was obtained from individual or guardian participants.

## Supporting information



Supporting Information

## Data Availability

The data that support the findings of this study are available on request from the corresponding author. The data are not publicly available due to privacy and ethical restrictions.
