# Supplementary material for: The Evolutionary Trajectory and Prognostic Value of GITR+ Tregs Reprogramed by Tumor‐Intrinsic PD‐1/c‐MET Signaling in Pancreatic Cancer
Source: Adv Sci (Weinh). 2025 Jul 17;12(36):e00806. doi: 10.1002/advs.202500806 (PMC12463103; doi:10.1002/advs.202500806)
Supplement: Supplementary file 1 — Supporting Information [file ADVS-12-e00806-s001.docx]

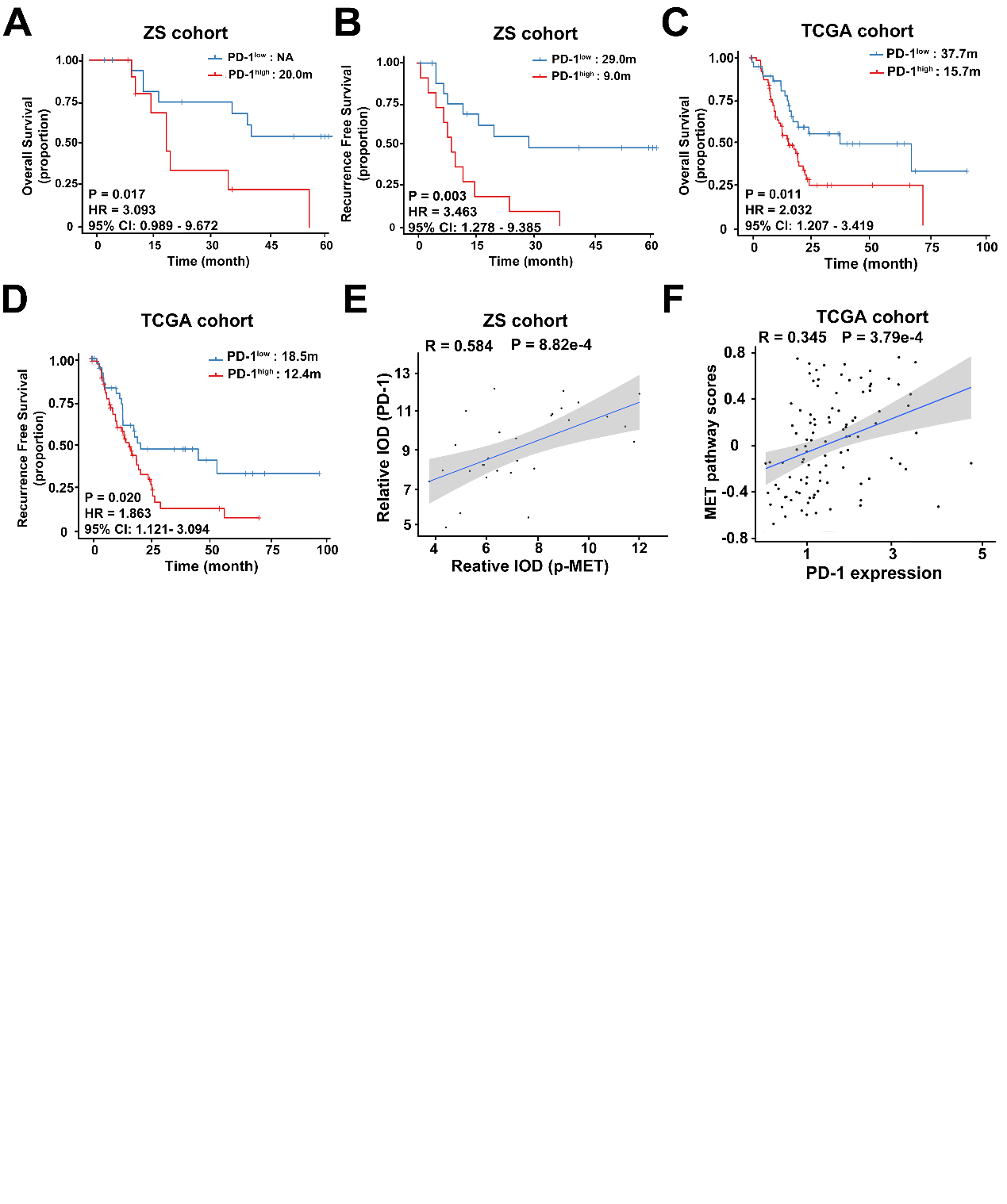


**Supplementary Figure 1**. **Tumor-intrinsic PD-1 is correlated with MET activation and acts as a predictor for PDAC prognosis**. (A, B) Kaplan-Meier curves for OS (A) and RFS (B) in PDAC patients with high or low tumor-intrinsic PD-1 expression in ZS cohort (n=29). (C, D) Kaplan-Meier curves for OS (C) and RFS (D) in PDAC patients from TCGA cohort with high or low tumor-intrinsic PD-1 expression. (E) Pearson correlation of p-MET and tumor intrinsic PD-1 relative IOD in ZS cohort (n=29). (F) Pearson correlation of MET activation and tumor-intrinsic PD-1 expression in PDAC patients from TCGA cohort. OS, overall survival; RFS, recurrence-free survival; IOD, integrated optical density; TCGA, The Cancer Genome Atlas; PDAC, pancreatic ductal adenocarcinoma; TMA, tissue microarray.


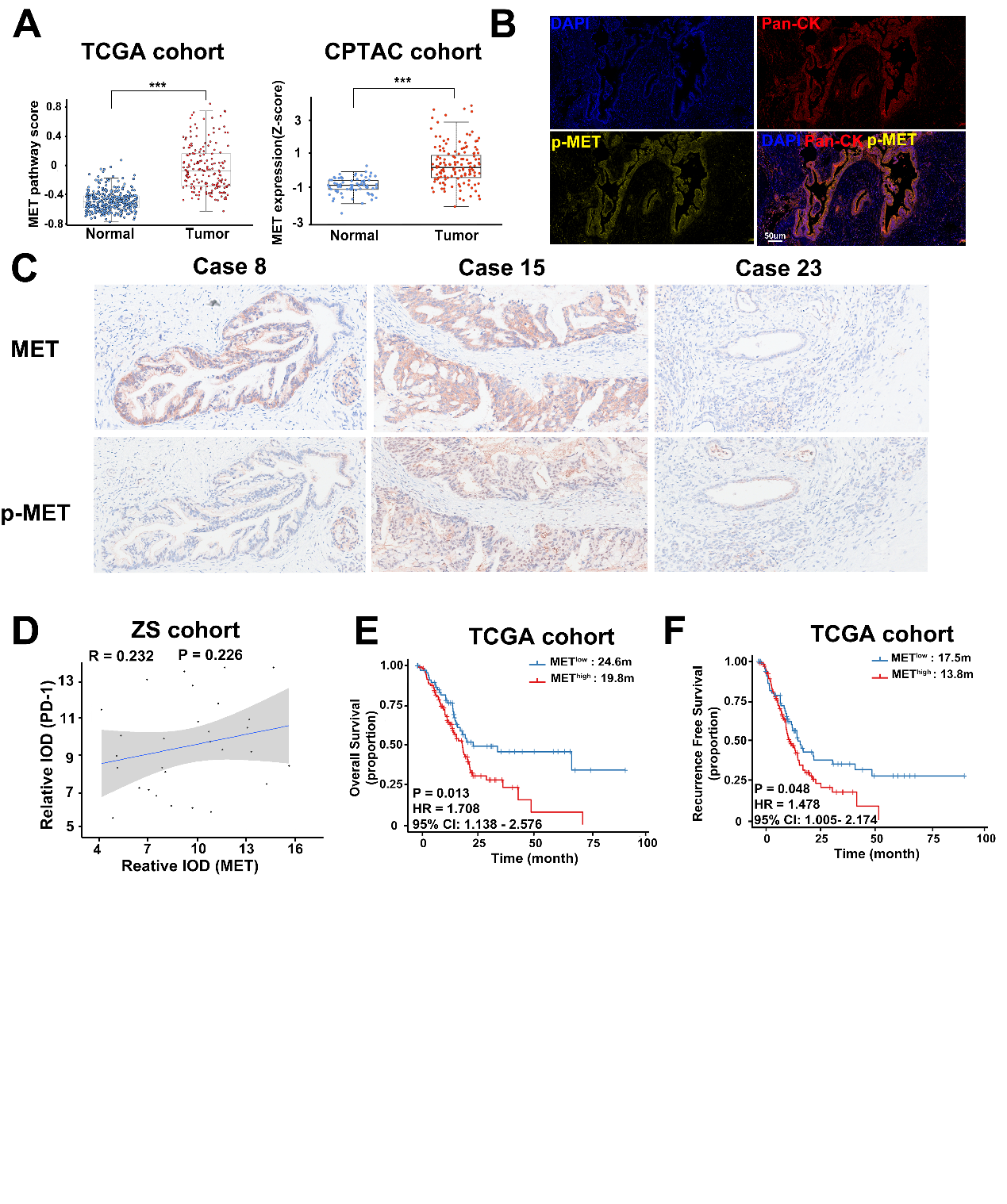


**Supplementary Figure 2. MET is upregulated in PDAC and associated with worse prognosis.** (A) MET activation score in PDAC and normal pancreas in PDAC patients from TCGA database (left) and MET protein level in PDAC patients from CTPAC database (right), ***p<0.001. (B) Representative immunofluorescence images for co-localization of pan-CK and p-MET. (C) Representative cases for high or low MET and p-MET expression. (D) Pearson correlation of MET and tumor intrinsic PD-1 relative IOD in ZS cohort (n=29). (E, F) Kaplan-Meier curves for OS (E) and RFS (F) in PDAC patients from TCGA cohort with high or low MET activation. OS, overall survival; RFS, recurrence-free survival; TCGA, The Cancer Genome Atlas; PDAC, pancreatic ductal adenocarcinoma.


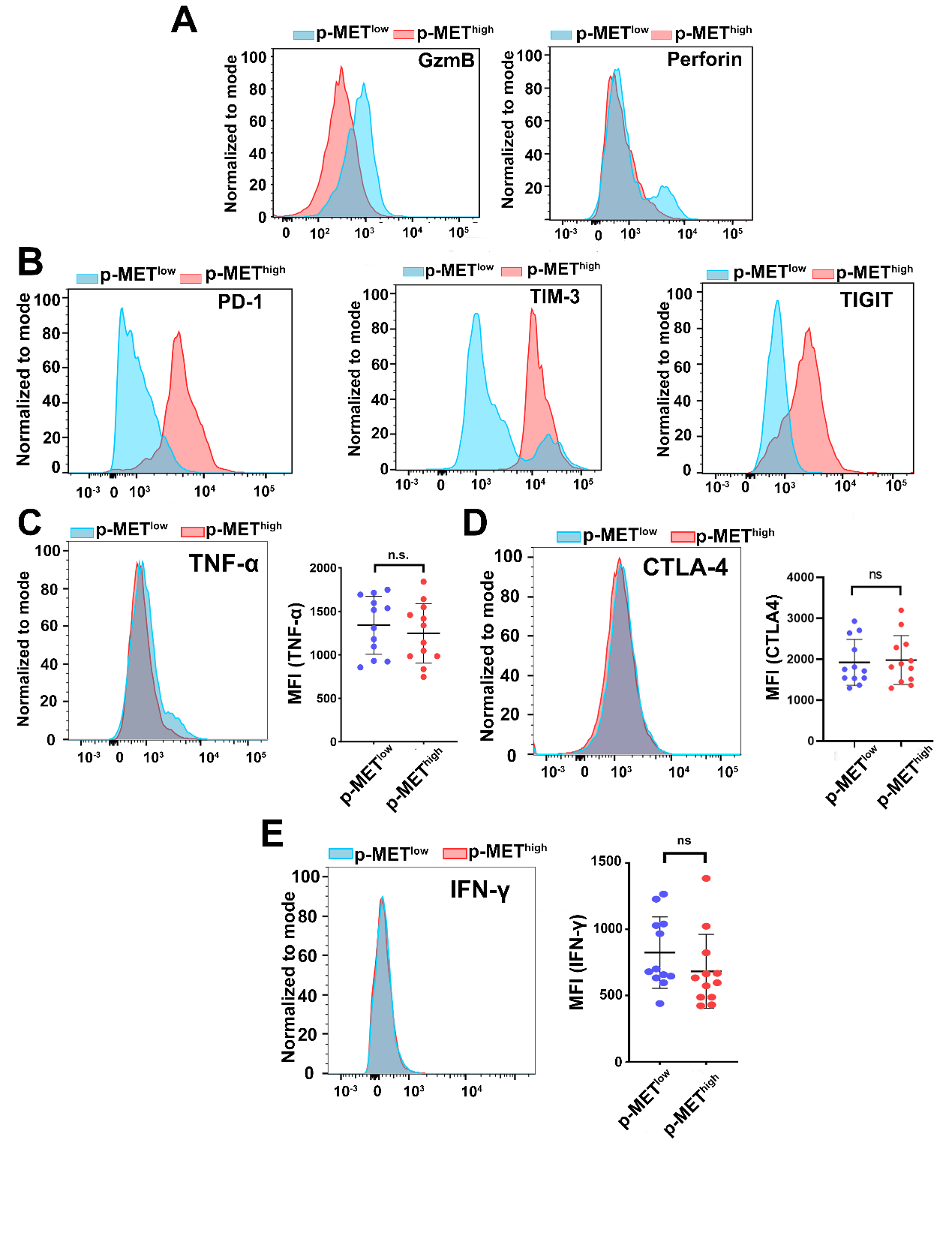


**Supplementary Figure 3. MET activation is associated with impaired CD8^+^ T cell function in PDAC tissues.** (A) Flow cytometry histogram displaying GzmB and Perforin expression in p-MET high or p-MET low tumors, see also in **Figure 1E**. (B) Flow cytometry histogram displaying PD-1, TIM-3 and TIGIT expression in p-MET high or p-MET low tumors, see also in **Figure 1F**. (C-E) Flow cytometry analysis of TNF-a (C), CTLA-4 (D) and IFN-γ (E) in p-MET high or p-MET low tumors. n=12 per group. ns, not significant.


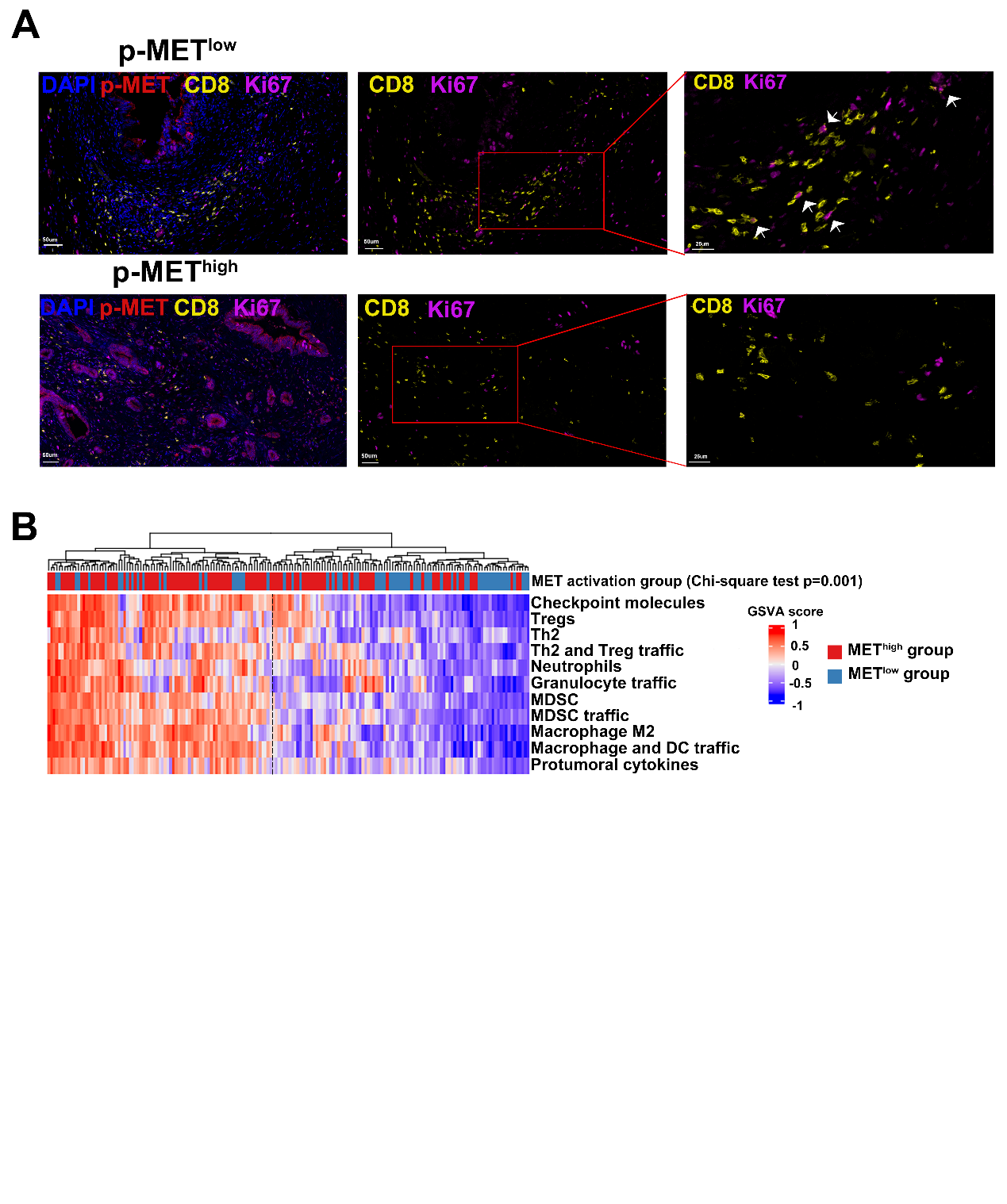

**Supplementary Figure 4. MET activation is associated with impaired CD8^+^ T cell proliferating and immunosuppressive TME.** (A) Representative immunofluorescence staining images for Ki67^+^CD8^+^ T cells in p-MET high or p-MET low tumors. (B) Heatmap displaying the relationship between MET pathway activation and immunosuppressive microenvironment in PDAC patients from TCGA cohort. TCGA, The Cancer Genome Atlas; PDAC, pancreatic ductal adenocarcinoma.


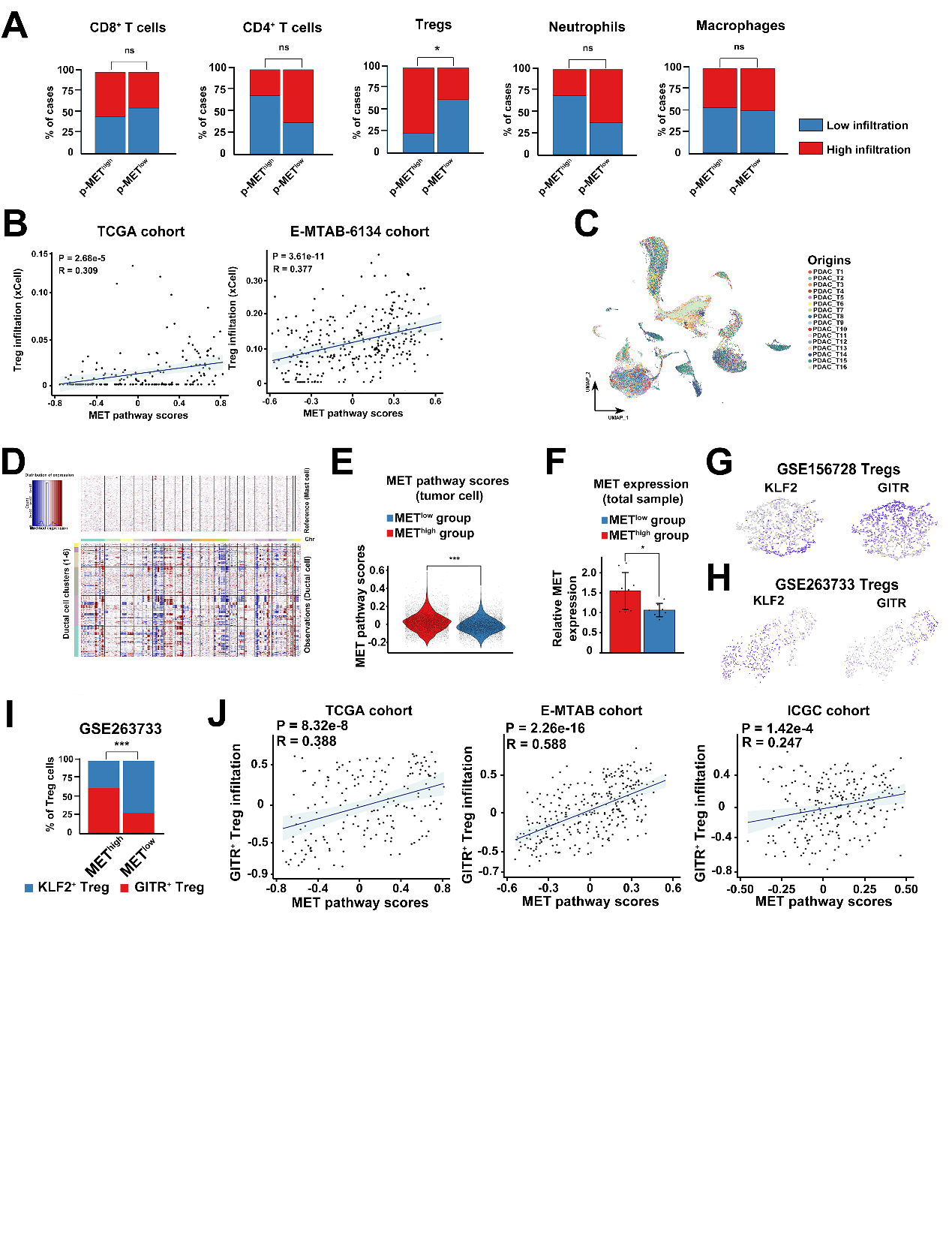


**Supplementary Figure 5**. **MET activation is positively correlated with GITR^+^ Tregs infiltration in PDAC.** (A) Percentages of PDAC specimens with high or low immune cells infiltration. ns, not significant, *p<0.05. (B) Pearson correlation of MET activation and Tregs infiltration in TCGA and E-MTAB-6134 cohorts. (C) UMAP plots of single cells identified by scRNA-seq and colored by different origins. (D) Copy number variations in different ductal cell clusters quantified by inferCNV algorithm. (E) MET pathway scores in MET^high^ and MET^low^ group at a single-cell level. ***p<0.001. (F) MET expression in MET^high^ and MET^low^ group at sample level. *p<0.05. (G, H) UMAP plots displaying GITR and KLF2 expression in Tregs from GSE156728 (G) and GSE263733 (H) datasets. (I) Percentages of GITR^+^ Tregs and KLF2^+^ Tregs in MET^high^ or MET^low^ group from GSE263733 datasets. ***p<0.001. (J) Pearson correlations of MET activation and GITR^+^ Treg infiltration in TCGA, E-MTAB-6134 and ICGC cohorts. UMAP, uniform manifold approximation and projection; TCGA, The Cancer Genome Atlas; ICGC, International Cancer Genome Consortium; PDAC, pancreatic ductal adenocarcinoma.


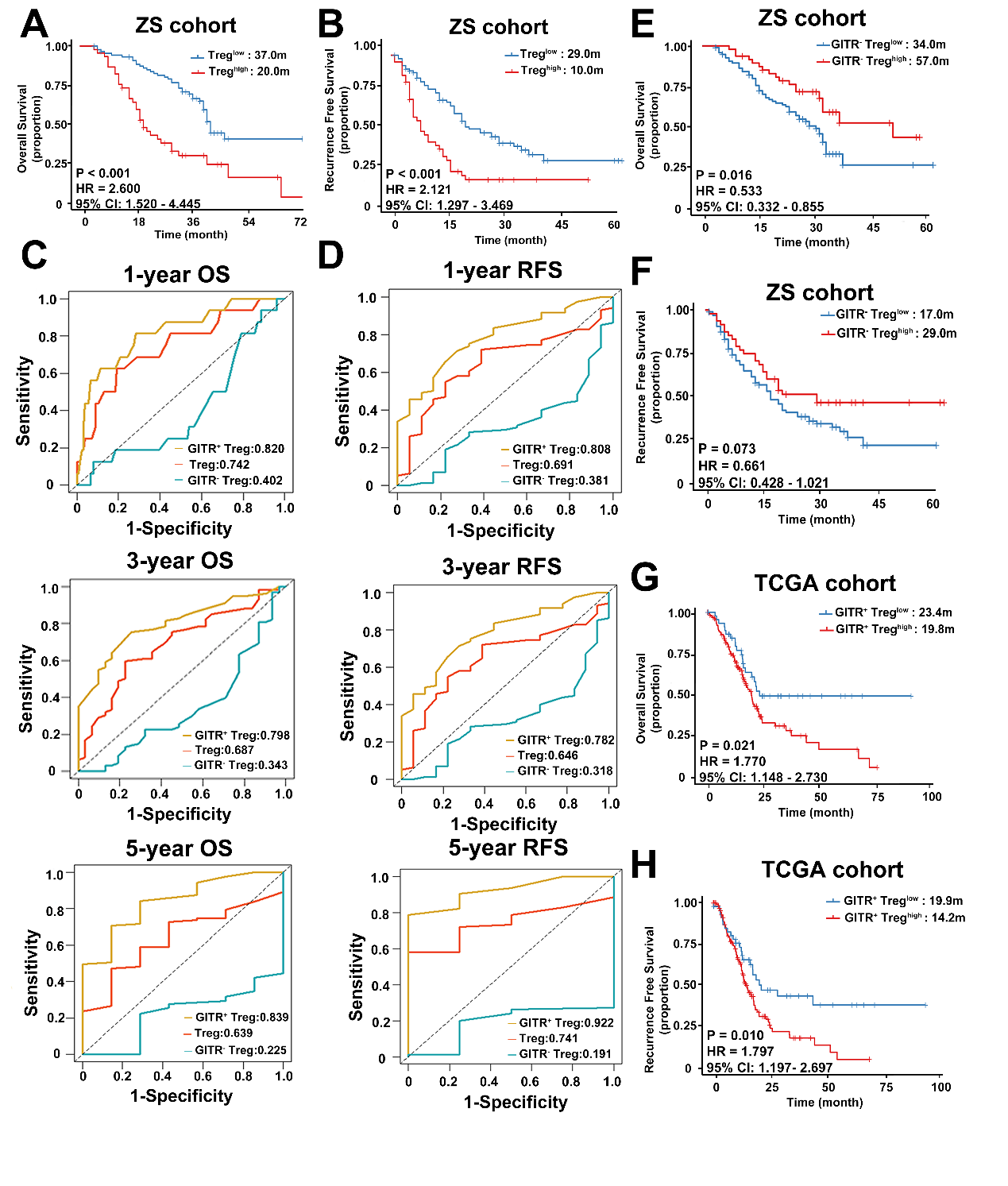


**Supplementary Figure 6**. **Survival analysis based on Tregs, GITR^-^ Tregs, and GITR^+^ Tregs infiltration.** (A, B) Kaplan-Meier curves for OS (A) and RFS (B) in PDAC patients with high or low Tregs infiltration in ZS cohort (n=145). (C, D) The 1-year, 3-year, and 5-year OS (C) and RFS (D) receiver operating characteristic (ROC) curve of Tregs, GITR^+^ Tregs and GITR^-^ Tregs. (E, F) Kaplan-Meier curves for OS (E) and RFS (F) in PDAC patients with high or low GITR^-^ Tregs infiltration in ZS cohort (n=145). (G, H) Kaplan-Meier curves for OS (G) and RFS (H) in PDAC patients with high or low GITR^+^ Tregs infiltration in TCGA cohort. OS, overall survival; RFS, recurrence-free survival; PDAC, pancreatic ductal adenocarcinoma; TCGA, The Cancer Genome Atlas.


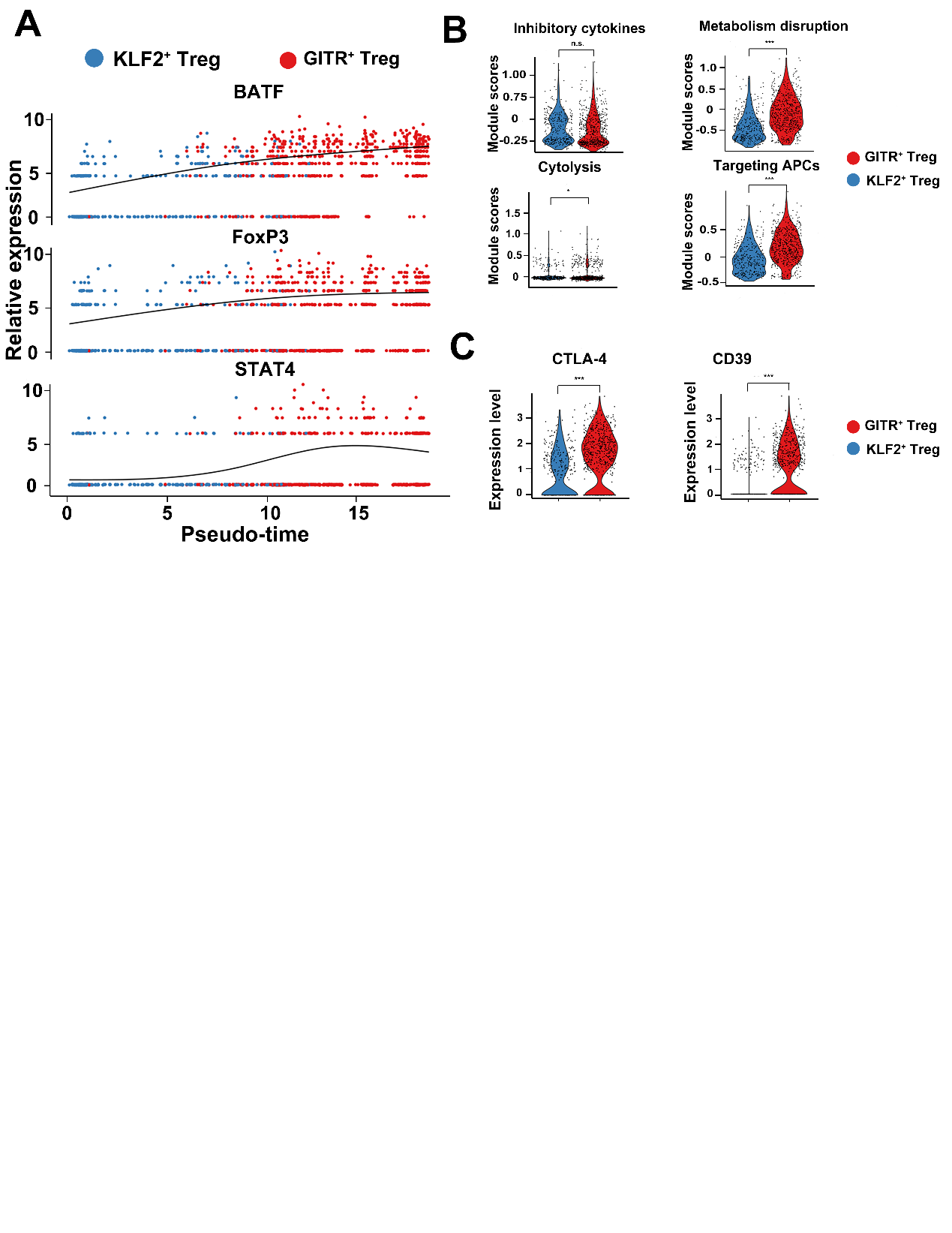


**Supplementary Figure 7. Tumor-infiltrating GITR^+^ Tregs as pronounced immunosuppressive effector Tregs in PDAC.** (A) Pseudo-time projections of transcriptional changes for Tregs transcription factor. (B) Module scores of inhibitory cytokines, metabolism disruption, cytolysis, and targeting APCs for GITR^+^ Tregs and KLF2^+^ Tregs from GSE155698. ns, not significant, *p<0.05, ***p<0.001. (C) Transcriptional expression of CTLA-4 and CD39 in GITR^+^ Tregs and KLF2^+^ Tregs from GSE155698. ***p<0.001.


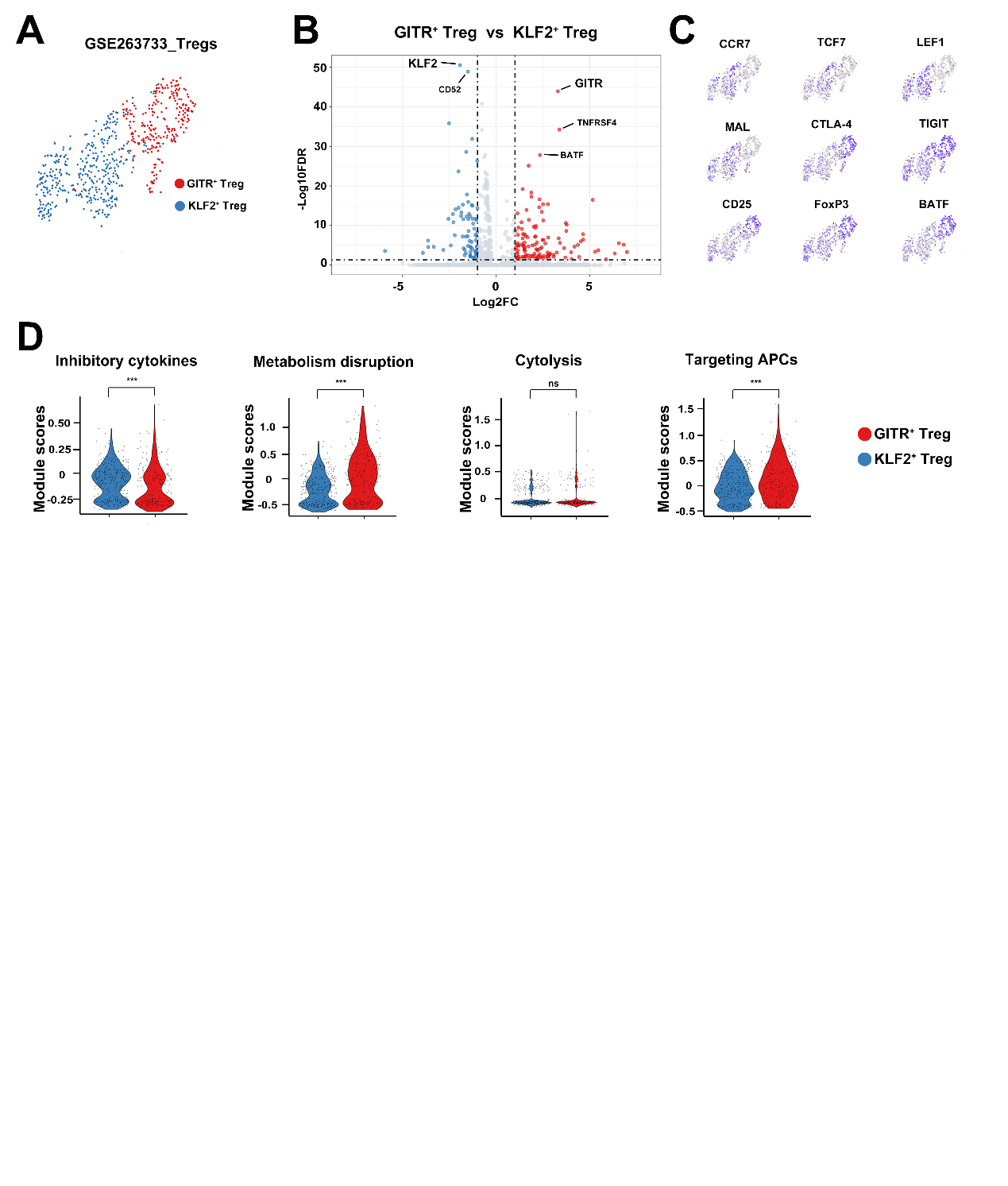


**Supplementary Figure 8. Validation of GITR^+^ Tregs immunosuppression in single cell RNA sequencing datasets.** (A) UMAP plots of Tregs from GSE263733 colored by different Tregs subclusters. (B) Differentially expressed genes between GITR^+^ Tregs and KLF2^+^ Tregs from GSE263733. (C) UMAP plots displaying immune genes expression in Tregs from GSE263733. (D) Module scores of inhibitory cytokines, metabolism disruption, cytolysis, and targeting APCs for GITR^+^ Tregs and KLF2^+^ Tregs from GSE263733. ns, not significant, ***p<0.001. UMAP, uniform manifold approximation and projection­­­.


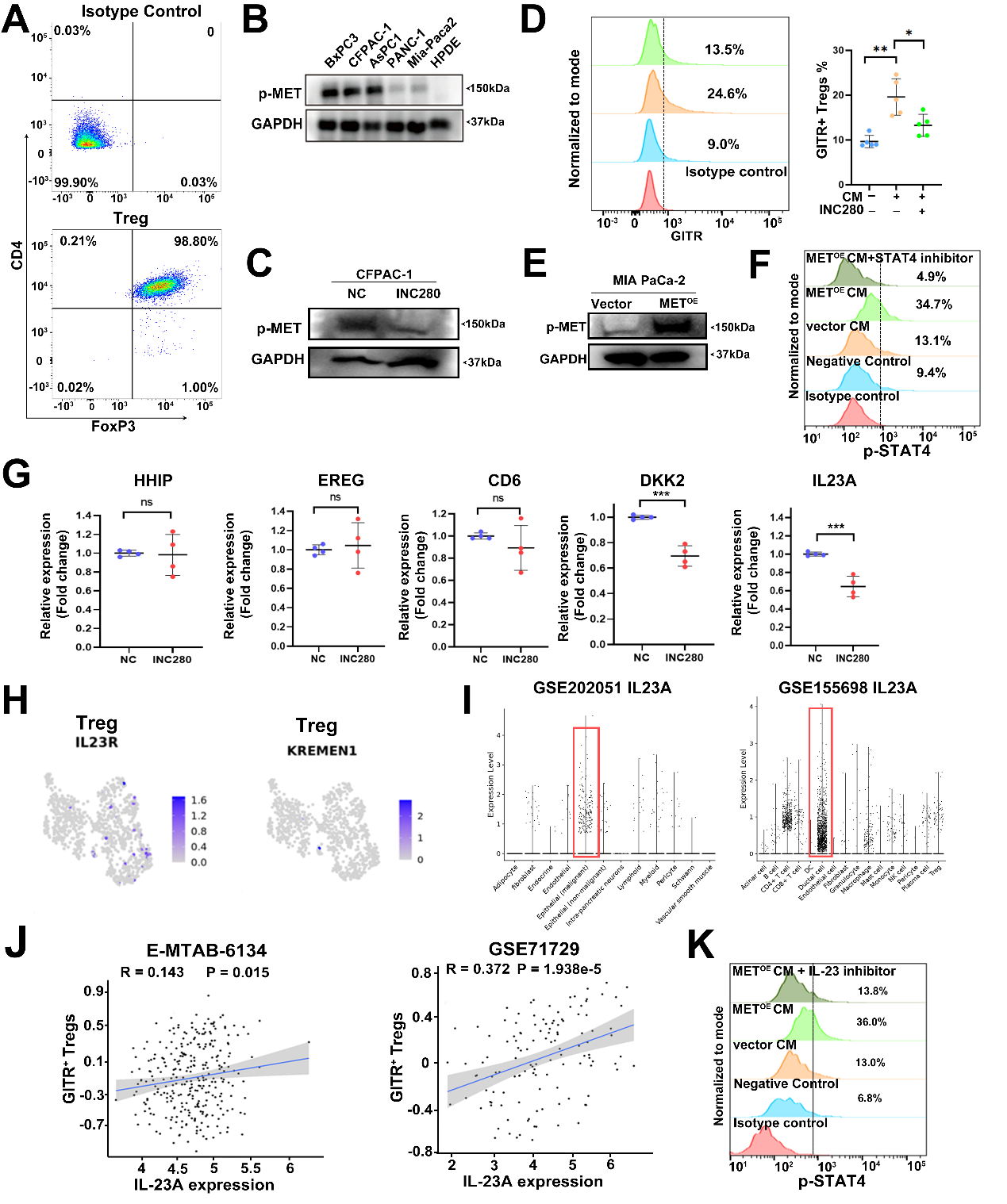


**Supplementary Figure 9. MET activation induces GITR^+^ Tregs accumulation through IL-23/STAT4 pathway.** (A) Flow cytometry validation of the Tregs purity based on CD4 and FoxP3 expression. (B) Western blot analysis of p-MET expression in different human PDAC cell lines and HPDE. (C) Western blot analysis of p-MET level in CFPAC-1 cells after INC280 treatment. (D) Flow cytometry analysis of GITR^+^ Tregs frequency with/without BxPC3 CM in the presence or absence of INC280. n=5 per group. *p<0.05, **p<0.01. (E) Western blot analysis of p-MET after overexpression in MIA-PaCa-2 cells. (F) Flow cytometry analysis of p-STAT4 level in Tregs with/without MIA-PaCa-2 vector/MET^OE^ CM in the presence or absence of STAT4 inhibitor. (G) qPCR validation of genes correlated with MET activation and GITR^+^ Tregs program. n=4 per group. ns, not significant, ***p<0.001. (H) UMAP plots displaying IL-23R and KREMEN1 expression in Tregs from GSE155698. (I) Violin plots displaying IL-23A expression in different cell types in PDAC. (J) Pearson correlations of GITR^+^ Tregs and IL-23A expression in E-MTAB-6134 cohort and GSE71729 cohort. (K) Flow cytometry analysis of p-STAT4 expression in Tregs with/without MIA-PaCa-2 vector/MET^OE^ CM in the presence or absence of IL-23 inhibitor. UMAP, uniform manifold approximation and projection­­­; PDAC, pancreatic ductal adenocarcinoma; TCGA, The Cancer Genome Atlas; WB, western blot.


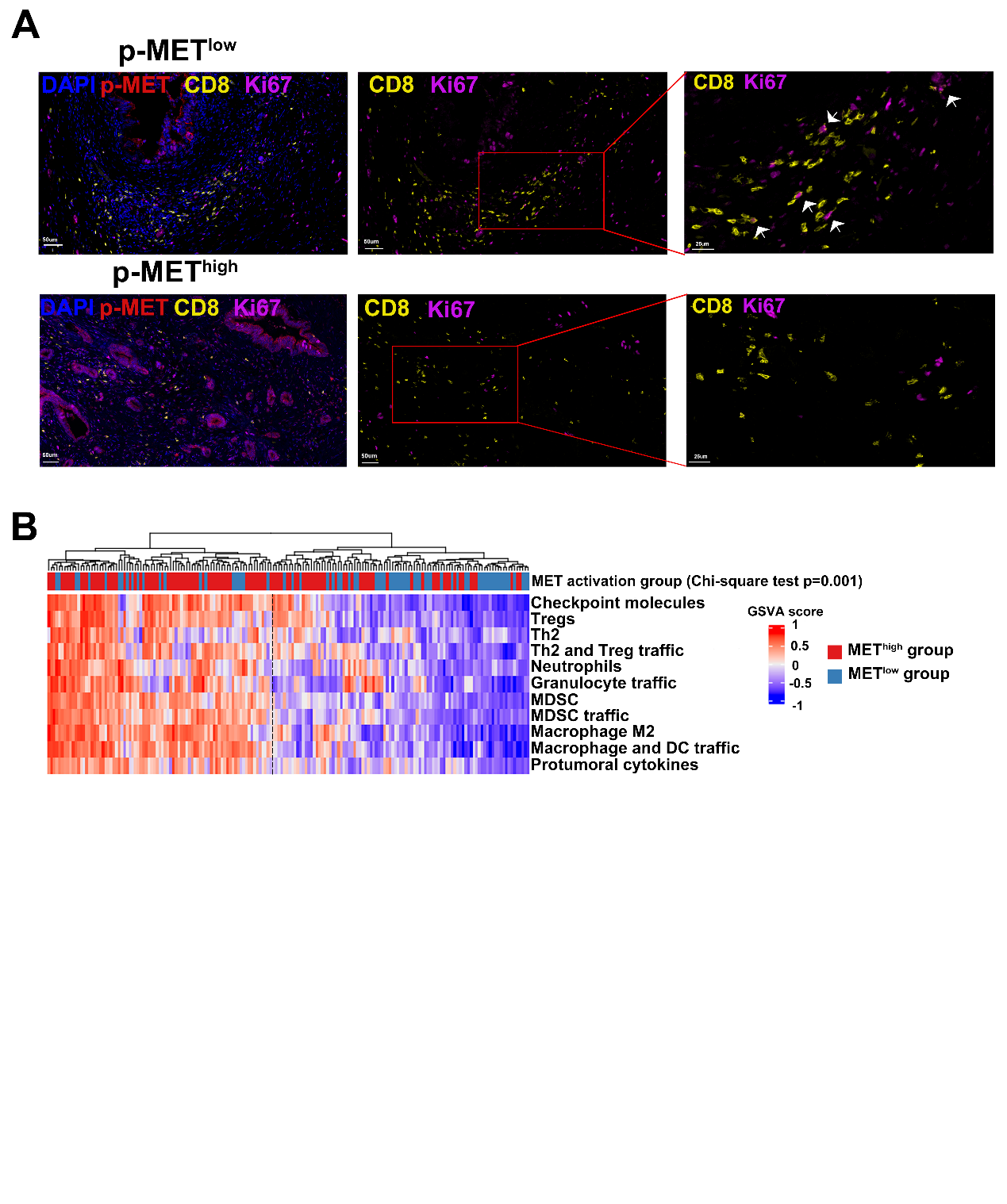


**Supplementary Table 1. The relationships between tumor MET expression and clinicopathological characteristics and tumor-infiltrating cells.**

| **Variables** | **Low MET** | **High MET** | ***P* value** |
| --- | --- | --- | --- |
|  | **(n=16)** | **(n=13)** |  |
| **Gender** |  |  | 0.752 |
| Male/ Female | 13/3 | 9/4 |  |
| **Age (years)** |  |  | 0.379 |
| <65/ ≥ 65 | 10/6 | 6/7 |  |
| **Tumor site** |  |  | 0.897 |
| Head/Body&Tail | 7/9 | 6/7 |  |
| **Differentiation** |  |  | 0.871 |
| I/ II/ III | 2/5/9 | 1/5/7 |  |
| **T stage** |  |  | 0.235 |
| T1/T2 | 14/2 | 8/5 |  |
| **N stage** |  |  | 0.340 |
| N0/N1+N2 | 9/7 | 5/8 |  |
| **TNM stage**  I/ II/ III | 7/8/1 | 3/5/5 | 0.095 |
| **CA19-9** |  |  | 0.774 |
| <200/≥200 U/mL | 9/7 | 8/5 |  |
| **CEA** |  |  | 1.000 |
| <5/≥5 U/mL | 12/4 | 10/3 |  |
| **CA125** |  |  | 0.235 |
| <25/≥25 U/mL | 14/2 | 8/5 |  |
| **ART**  No / Yes | 13/3 | 12/1 | 0.751 |
| **ACT Regimen** |  |  | 1.000 |
| Gemcitabine based/Others | 12/4 | 10/3 |  |
| **Adipose infiltration** |  |  | 1.000 |
| No / Yes | 2/14 | 2/11 |  |
| **Microvascular invasion** |  |  | 1.000 |
| No / Yes | 10/6 | 9/4 |  |
| **Perineural invasion** |  |  | 0.273 |
| No / Yes | 5/11 | 1/12 |  |
| **PD-1 expression**  Low/ High | 13/3 | 5/8 | **0.048** |
| **Tumor CD8 T cells**  Low/ High | 9/7 | 7/6 | 0.897 |
| **Tumor CD4 T cells**  Low/ High | 6/10 | 9/4 | 0.089 |
| **Tumor Tregs**  Low/ High | 10/6 | 3/10 | **0.034** |
| **Tumor Neutrophils**  Low/ High | 6/10 | 9/4 | 0.089 |
| **Tumor Macrophages**  Low/ High | 8/8 | 7/6 | 0.873 |

**Supplementary Table 2. Univariate and multivariate analysis of prognostic indicators associated with overall survival and recurrence-free survival.**

| **Variables** | **Overall survival** | | | **Recurrence-free survival** | | |
| --- | --- | --- | --- | --- | --- | --- |
|  | **Univariate *P* value** | **Multivariate *P* value** | **Multivariate HR (95% CI)** | **Univariate *P* value** | **Multivariate *P* value** | **Multivariate HR (95% CI)** |
| **Gender** |  |  |  |  |  |  |
| Male/Female | 0.250 | NA |  | 0.315 | NA |  |
| **Age (years)** |  |  |  |  |  |  |
| <65/≥65 | 0.315 | NA |  | **0.017** | 0.081 | 2.589 (0.890-7.530) |
| **Tumor site** |  |  |  |  |  |  |
| Head/Body&Tail | 0.110 | NA |  | 0.193 | NA |  |
| **Differentiation** |  |  |  |  |  |  |
| I/II/III | 0.333 | NA |  | 0.797 | NA |  |
| **T stage** |  |  |  |  |  |  |
| T1/T2 | 0.425 | NA |  | 0.806 | NA |  |
| **N stage** |  |  |  |  |  |  |
| N0/N1+N2 | 0.187 | NA |  | 0.408 | NA |  |
| **TNM stage** |  |  |  |  |  |  |
| I/II/III | 0.084 | NA |  | 0.410 | NA |  |
| **CA19-9** |  |  |  |  |  |  |
| <200/≥200 U/mL | 0.805 | NA |  | 0.739 | NA |  |
| **CEA** |  |  |  |  |  |  |
| <5/≥5 U/mL | 0.641 | NA |  | 0.667 | NA |  |
| **CA125** |  |  |  |  |  |  |
| <25/≥25 U/mL | 0.102 | NA |  | 0.474 | NA |  |
| **ART** |  |  |  |  |  |  |
| No / Yes | 0.607 | NA |  | 0.966 | NA |  |
| **ACT Regimen** |  |  |  |  |  |  |
| Gemcitabine based/Others | 0.255 | NA |  | 0.529 | NA |  |
| **Adipose infiltration** |  |  |  |  |  |  |
| No / Yes | 0.373 | NA |  | 0.945 | NA |  |
| **Microvascular invasion** |  |  |  |  |  |  |
| No / Yes | 0.795 | NA |  | 0.463 | NA |  |
| **Perineural invasion** |  |  |  |  |  |  |
| No / Yes | 0.139 | NA |  | 0.206 | NA |  |
| **MET expression**  Low/ High | **0.025** | **0.025** | 3.604 (1.173-11.075) | **0.007** | **0.031** | 3.032 (1.109-8.291) |

**Supplementary Table 3. The clinicopathological characteristics in the total Zhongshan cohort.**

| **Characteristics** | **Patients (n=145)** |
| --- | --- |
| **Sex** |  |
| Male/Female | 91/54 |
| **Age(years)** |  |
| <65/≥65 | 68/77 |
| **Differentiation** |  |
| I/II/III | 6/76/63 |
| **T stage** |  |
| T1/T2/T3 | 46/78/21 |
| **N stage** |  |
| N0/N1/N2 | 83/52/10 |
| **TNM stage** |  |
| I/II/III | 71/64/10 |
| **ART** |  |
| No/Yes | 105/40 |
| **ACT** |  |
| No/Yes | 16/129 |
| **Microvascular invasion** |  |
| No/Yes | 104/41 |
| **Perineural invasion** |  |
| No/Yes | 22/123 |
| **CA19-9** |  |
| Median (IQR) | 80.6 (19.5-356.9) |
| **CEA** |  |
| Median (IQR) | 3.2 (2.2-5.3) |
| **Tumor-infiltrating GITR+ Tregs** |  |
| Median (IQR) | 4.7 (2.7-8.3) |
| **Tumor-infiltrating Tregs** |  |
| Median (IQR) | 10.6 (7.7-13.8) |

ART, adjuvant radiation therapy; ACT, adjuvant chemotherapy; Treg, regulatory T cell; IQR, interquartile range.

**Supplementary Table 4. Primers for RT-qPCR.**

|  | Forward primer | Reverse primer |
| --- | --- | --- |
| Human IL23A | CACTAGTGGGACACATGGATCT | AGAGAAGGCTCCCCTGTGAA |
| Human HHIP | AATGCAGAGCCACGGTACAA | GCTGGCTCACATTTTGCAGT |
| Human CD6 | CAACAGTGAGTCGAGCACCT | GCTGGGGGACCCTGAAAA |
| Human DKK2 | CTCTGGATGGTACTCGGCAC | CTCTGGATGGTACTCGGCAC |
| Human EREG | CCTGGTGCACAGTGCTTAGA | GACCATAGACTCCCCAGGGT |

RT-qPCR, Real-time Quantitative Polymerase Chain Reaction.­
